# SIRT1 Undergoes Alternative Splicing in a Novel Auto-Regulatory Loop with p53

**DOI:** 10.1371/journal.pone.0013502

**Published:** 2010-10-21

**Authors:** Cian J. Lynch, Zahid H. Shah, Simon J. Allison, Shafiq U. Ahmed, Jack Ford, Lorna J. Warnock, Han Li, Manuel Serrano, Jo Milner

**Affiliations:** 1 YCR p53 Research Unit, Department of Biology, University of York, York, United Kingdom; 2 Tumour Suppression Group, Molecular Oncology Program, Spanish National Cancer Research Centre (CNIO), Madrid, Spain; Roswell Park Cancer Institute, United States of America

## Abstract

**Background:**

The NAD-dependent deacetylase SIRT1 is a nutrient-sensitive coordinator of stress-tolerance, multiple homeostatic processes and healthspan, while p53 is a stress-responsive transcription factor and our paramount tumour suppressor. Thus, SIRT1-mediated inhibition of p53 has been identified as a key node in the common biology of cancer, metabolism, development and ageing. However, precisely how SIRT1 integrates such diverse processes remains to be elucidated.

**Methodology/Principal Findings:**

Here we report that SIRT1 is alternatively spliced in mammals, generating a novel SIRT1 isoform: SIRT1-ΔExon8. We show that SIRT1-ΔExon8 is expressed widely throughout normal human and mouse tissues, suggesting evolutionary conservation and critical function. Further studies demonstrate that the SIRT1-ΔExon8 isoform retains minimal deacetylase activity and exhibits distinct stress sensitivity, RNA/protein stability, and protein-protein interactions compared to classical SIRT1-Full-Length (SIRT1-FL). We also identify an auto-regulatory loop whereby SIRT1-ΔExon8 can regulate p53, while in reciprocal p53 can influence SIRT1 splice variation.

**Conclusions/Significance:**

We characterize the first alternative isoform of SIRT1 and demonstrate its evolutionary conservation in mammalian tissues. The results also reveal a new level of inter-dependency between p53 and SIRT1, two master regulators of multiple phenomena. Thus, previously-attributed SIRT1 functions may in fact be distributed between SIRT1 isoforms, with important implications for SIRT1 functional studies and the current search for SIRT1-activating therapeutics to combat age-related decline.

## Introduction

From yeast to humans, SIRT1 is a highly conserved protein deacetylase and epigenetic sculptor of chromatin characteristics [1]–[5]. SIRT1 also modulates multiple signaling factors. Thus SIRT1 acts at several levels in concert to influence stress tolerance, homeostasis, cell proliferation, circadian rhythm, differentiation, development, and longevity [1]–[10]. Precisely how the SIRT1 protein governs so many processes has become the subject of intense investigation. Importantly, SIRT1 couples target substrate deacetylation with cellular metabolic status via its dependence on levels of NAD^+^, a co-enzyme and metabolic intermediate indicative of intracellular energy flux and redox potential [1]–[4], [7]. Recently, manipulation of SIRT1 activity has profoundly influenced in vivo models of obesity, neuro-degeneration, diabetes, cancer and aging [1]–[5], [11], [12]. Moreover, mammalian SIRT1 knockout is typically embryonic lethal, with surviving mice displaying severe developmental abnormalities including exencephaly, sterility and heart/retinal defects [5], [6], [8].

The tumour suppressor p53 is mutated, deleted or indirectly inactivated in the majority of human cancers, illustrating its importance as ‘guardian of the genome’ [13]–[15]. In response to diverse genotoxic stimuli, p53 instigates transcriptional programs and protein-protein interactions which orchestrate DNA repair, cell-cycle arrest, senescence or apoptosis, as appropriate [13]–[15]. p53 also influences key developmental decisions through pathways which remain to be elucidated: the p53-dependent stress-response is highly sensitive throughout embryonic tissues and p53 knockout mice reveal embryonic abnormalities such as exencephaly [16]–[18]. Indeed, p53 acts as a fundamental barrier to cell immortalization, blocking not only cancer-associated hyper-proliferation, but also limiting de-differentiation of cells towards induced pluripotency [19], [20]. Similar to SIRT1, p53 has also recently been shown to regulate metabolism in numerous ways [21]. However, basal p53 expression levels are low and limiting for function [16], [22]. Crucially therefore, considerable p53 accumulation is required to efficiently execute p53-dependent functions. Modifying p53 function in-vivo has profound outcomes: constitutive p53 hypo- or hyper-activity adversely affects lifespan by increasing cancer incidence or accelerating ageing respectively [23]–[26]. However, elevating p53 expression levels while maintaining normal regulation of its activity can improve cancer resistence and delay aging [27]–[29]. Thus p53 levels and activity are fine-tuned by evolution to achieve the optimal balance between cancer-resistance and longevity.

Acetylation represents an important p53 post-translational activation signal [30]. SIRT1 deacetylates and thereby represses p53 activity to protect cells from p53-dependent anti-proliferative responses [31], [32]. SIRT1 and p53 exert powerful and often opposing influences in multiple overlapping processes, and therefore, the SIRT1-p53 relationship may represent a key node in the common biology of cancer, metabolism, development and ageing [31]–[33].

Here we report that human SIRT1 is alternatively spliced in a manner which is stress-sensitive, p53-dependent and conserved in mammals. The novel SIRT1 isoform, SIRT1-ΔExon8, displays significant differences in stress-sensitivity, RNA/protein stability, protein-protein interactions and deacetylase activity compared to classical SIRT1-FL. The existence of alternative SIRT1 isoforms with distinct characteristics provides insight into the complex role of SIRT1 in vivo.

## Results

### SIRT1 is alternatively spliced, thereby amplifying its functional potential

**Figure 1:**
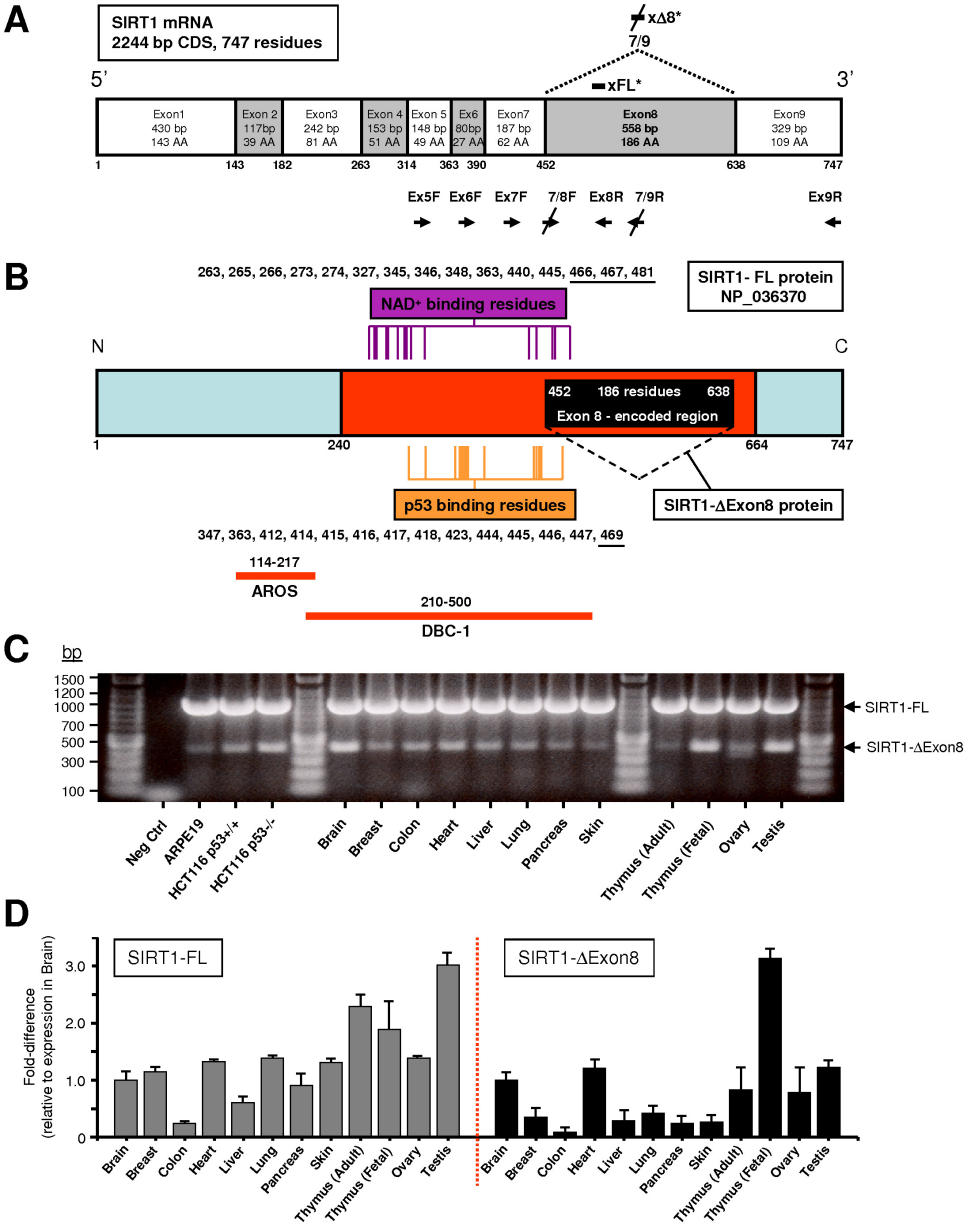
Identification of a novel SIRT1 splice variant which is expressed widely in human tissues. (**A**) Human SIRT1 splice-variant specific PCR primers and siRNA. Schematic displays human SIRT-FL coding mRNA sequence (based on NM_012238.3, GI: 13775598) plus loci of primers used (arrows, below panel). Primers were used in pairs in a RT-PCR-based tiling approach to identify any Exon-skipping SIRT1 transcripts. The novel Exon7/9 splice junction and the target loci of siRNA which selectively target SIRT1-ΔExon8 (xΔ8*) or SIRT1-FL (xFL*) are shown above panel. Exon8 is the largest SIRT1 coding Exon and translation of SIRT1-Exon9 remains in-frame in the SIRT1-ΔExon8 transcript. For human primer and siRNA sequences see Figures S13 and S14. (**B**) Schematic of the 747-residue human SIRT1-Full-Length protein (SIRT1-FL; based on NP_036370) with the Exon8-encoded region highlighted (558bp, 186 residues, black) within the catalytic deacetylase domain (240-664, red) as defined by Sinclair and colleagues [2]. The conserved amino acid residues reported to be involved in NAD^+^-binding [48] and p53-binding [47] by SIRT1 homologues are listed and their relative loci are indicated by vertical lines. The SIRT1-ΔExon8 protein (561 residues) lacks a central part of the catalytic domain (186 amino acids), including three residues involved in NAD^+^-binding (underlined: 466, 467, 481). This is predicted to significantly alter NAD^+^-binding and deacetylase catalytic efficiency of SIRT1-ΔExon8. Note that only 1 of the 14 residues mediating the p53-SIRT1 interaction lies within the Exon8-encoded region and is therefore absent in the SIRT1-ΔExon8 protein (residue 469, underlined). The protein-binding regions for AROS and DBC-1 on SIRT1-FL have been defined [44], [45] and are indicated below the protein map. (**C**) Relative abundance of SIRT1-ΔExon8 (lower arrow) or SIRT1-FL (upper arrow) across a range of normal human tissues. RT-PCR co-amplification of the two transcripts was performed with 250 ng total RNA from each tissue and human primer pair Ex7F (Fwd) and Ex9R (Rvs) as in (A) above. (**D**) SIRT1-FL versus SIRT1-ΔExon8 expression across a range of normal human tissues. Splice-variant-specific quantitative Real-Time PCR (qRT-PCR) of SIRT1-FL or SIRT1-ΔExon8 is shown (Methods), with respect to expression levels in brain.

We hypothesised that SIRT1 mRNA might undergo splice variation to generate diverse isoforms of distinct purpose. This was assessed in human cells using multiple pairs of primers in a RT-PCR-based tiling approach (Figure 1A), which revealed a novel SIRT1 transcript skipping precisely the 558 bp of Exon8 only, to produce an Exon7/9 splice junction (Figures 1A and S1), as confirmed by sequencing the RT-PCR products (data not shown). SIRT1-ΔExon8 lacks a portion of the reported deacetylase catalytic domain [2] and is therefore predicted to exhibit significantly altered deacetylase activity and NAD^+^-binding efficiency compared to classical SIRT1-Full-Length (SIRT1-FL; Figures 1A and 1B). The SIRT1-ΔExon8 splice variant was ubiquitously expressed in all 12 normal human tissues tested, and in a panel of 16 human epithelial cell lines of normal- or cancer-origin (Figures 1C, 1D and S2); although SIRT1-FL was more abundant in each case. However, the relative abundance of the two transcripts showed significant tissue-specific variability. For example, prominent SIRT1-ΔExon8 expression was detected particularly in human brain, heart, fetal thymus and testis (Figure 1D). It is notable that multiple SIRT1 functions have previously been identified for neurons, cardiomyocytes and gametogenesis [3]–[6], [8]. The abundance of SIRT1-ΔExon8 also differed sharply between fetal thymus versus adult thymus, suggesting a developmental purpose, whereas SIRT1-FL abundance was equivalent in fetal and adult thymus (Figure 1D).

**Figure 2:**
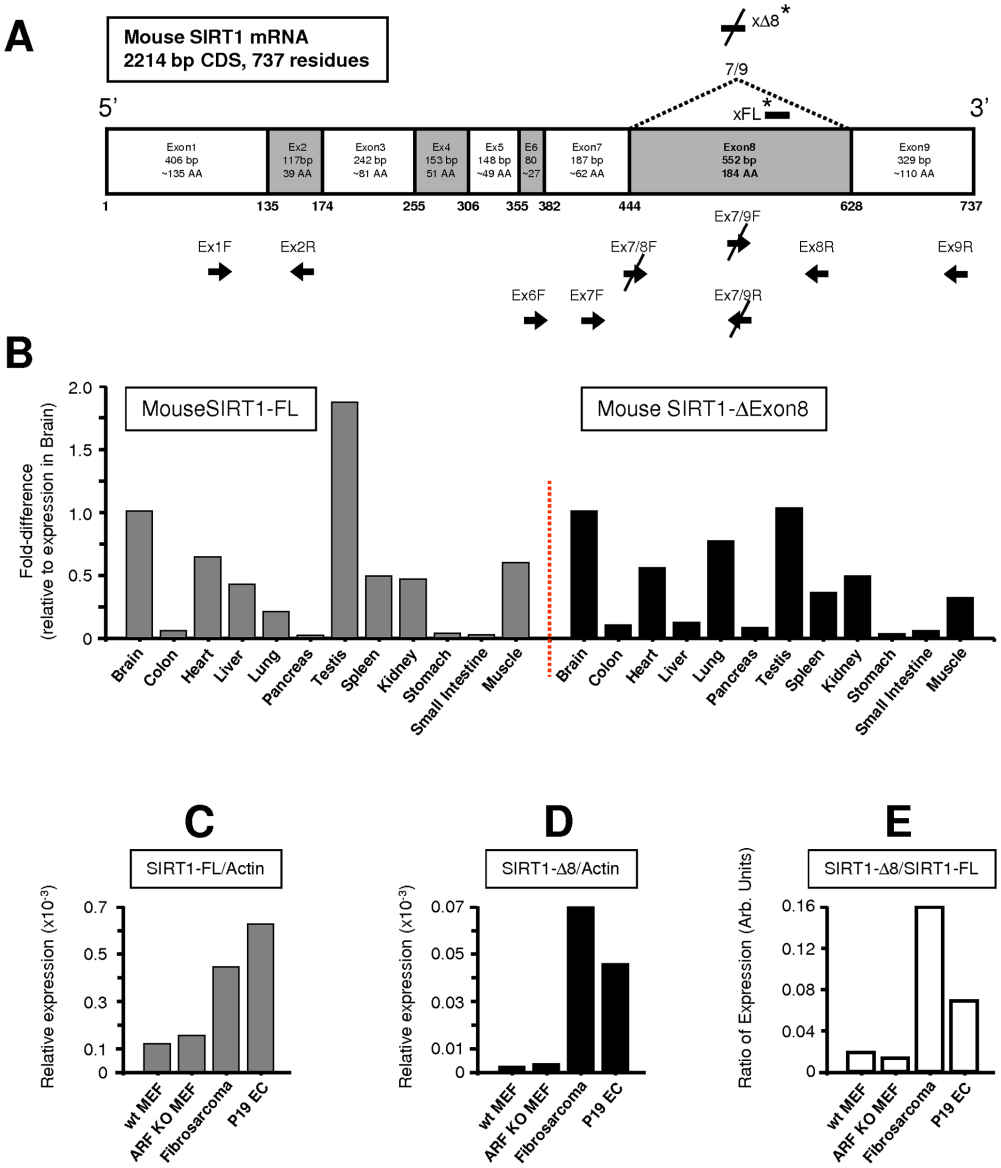
SIRT1-ΔExon8 is expressed widely in mouse cells and tissues. (**A**) Mouse SIRT1 splice-variant-specific PCR primers and siRNA. Schematic of mouse SIRT-FL coding mRNA sequence (737 residues; based on NM_019812.2, GI: 227430307) plus loci of primers used (arrows, below panel). The novel Exon7/9 splice junction and target loci of siRNA which selectively target mouse SIRT1-ΔExon8 (xΔ8*) or mouse SIRT1-FL (xFL*) are shown above panel. Similar to humans, mouse SIRT1 Exon8 is the largest coding Exon and translation of SIRT1-Exon9 remains in-frame in the mouse SIRT1-ΔExon8 transcript, generating a 553 residue protein. For mouse primer and siRNA sequences see Figures S13 and S15. (**B**) SIRT1-FL versus SIRT1-ΔExon8 expression across a range of normal mouse tissues. Mouse splice-variant-specific qRT-PCR of SIRT1-FL or SIRT1-ΔExon8 with respect to β-Actin using the delta-C(t) method (see Methods). Data is shown with respect to expression levels in brain. (**C**) Expression of SIRT1-FL in a panel of mouse cell lines. Mouse splice-variant-specific qRT-PCR was performed for SIRT1-FL and corrected by β-Actin levels as in (B) above. (**D**) Expression of SIRT1-ΔExon8 in a panel of mouse cell lines. Mouse splice-variant-specific qRT-PCR was performed for SIRT1-ΔExon8 and corrected by β-Actin levels as in (B) above. (**E**) Relative expression levels of SIRT1-FL versus SIRT1-ΔExon8 transcripts in a panel of mouse cell lines. The data in (C) and (D) above, which represents the relative expression of SIRT1-FL or SIRT1-ΔExon8 with respect to β-Actin by the delta-C(t) method, was used here to calculate the true relative ratio of SIRT1-ΔExon8/SIRT1-FL.

Transcript-specific RT-PCR analysis also demonstrated SIRT1-ΔExon8 expression in Mouse Embryonic Fibroblasts (MEFs)(Figures 2A and S3). Ubiquitous expression of the SIRT1-ΔExon8 splice variant was evident in a panel of 12 normal mouse tissues, and in mouse cell lines of normal- or cancer-origin (Figures 2B–2E). Similar to the pattern in human tissues, higher levels of SIRT1-ΔExon8 were present in mouse brain, heart and testis (compare: Figure 1D with 2B). Overall, the expression profile of SIRT1-ΔExon8 was comparable in mouse and human tissues, suggesting evolutionary conservation.

### SIRT1-FL and SIRT1-ΔExon8 display distinct characteristics

**Figure 3:**
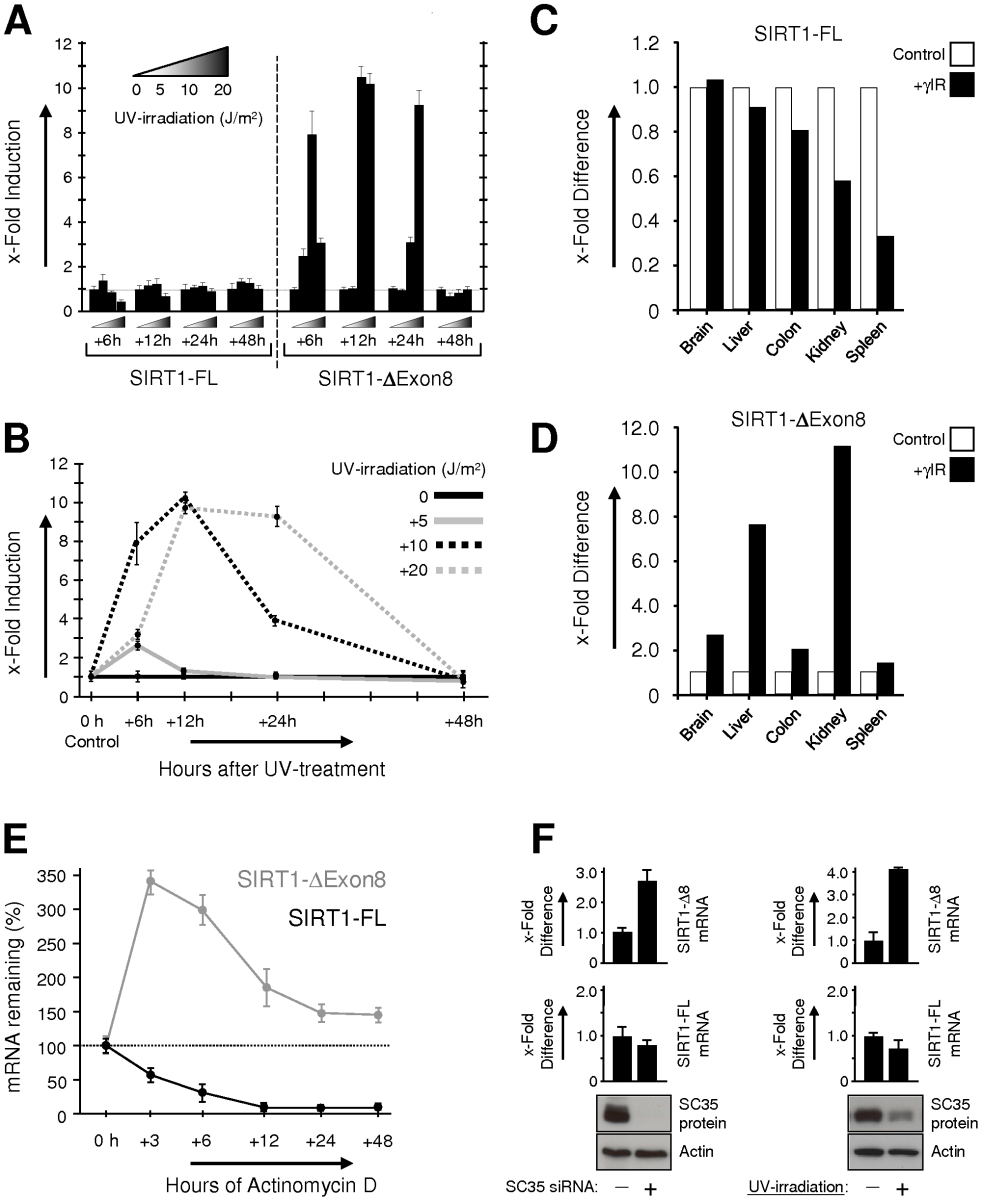
In contrast to SIRT1-FL, SIRT1-ΔExon8 displays stress-sensitivity and significant RNA stability. (**A**) Splice-variant-specific qRT-PCR was performed as in Figure 1 for SIRT1-FL or SIRT1-ΔExon8 mRNA levels in HCT116 p53+/+ cells, +/- UV stress treatment as indicated (see: Methods). (**B**) The qRT-PCR data in (A) above was plotted to highlight the dose-dependent pulse of SIRT1-ΔExon8 mRNA induction after stress. (**C**) SIRT1-FL mRNA levels in response to stress in vivo. Mouse SIRT1-FL mRNA expression was analysed by qRT-PCR and expressed relative to β-Actin by the delta-C(t) method in a panel of mouse tissues. Total RNA was harvested 2.5 hours after mock or whole-body γ-irradiation with 5 Gy. Data is shown with respect to levels in control mice for each tissue. (**D**) SIRT1-ΔExon8 mRNA levels display stress-induction in vivo. Mouse SIRT1-ΔExon8 mRNA expression was analysed by qRT-PCR and expressed relative to β-Actin by the delta-C(t) method in a panel of mouse tissues which were treated as in (C) above. Data is shown with respect to levels in control mice for each tissue. (**E**) Transcript stability of SIRT1-FL versus SIRT1-ΔExon8. qRT-PCR specific for SIRT1-FL or SIRT1-ΔExon8 was performed as above at intervals following transcriptional inhibition using Actinomycin D (see Methods). Initial expression levels of each transcript  = 100%. Results shown are for HCT116 p53−/− cells. Similar results were observed for HCT116 p53+/+, p53+/− and ARPE19 cells (data not shown). (**F**) Down-regulation of the SC35 splicing factor correlates with a selective increase in SIRT1-ΔExon8 expression. On the left, siRNA-mediated SC35 protein knockdown was performed in HCT116 human cells and verified by Western blot, followed by transcript-specific qRT-PCR for SIRT1-FL or SIRT1-ΔExon8. On the right, HCT116 cells were exposed to 10 J/m^2^ UV-irradiation and harvested 6 hours later. SC35 protein levels and qRT-PCR for SIRT1-FL or SIRT1-ΔExon8 were quantified as above.

In contrast to SIRT1-FL, SIRT1-ΔExon8 expression was sharply stress-inducible (Figures 3A and S4). UV-irradiation dose-titration and time-course analysis revealed a rapid and dose-dependent pulse of SIRT1-ΔExon8 expression (Figures 3A, 3B and S4). Pulse duration and amplitude were extended with increasing stress severity, and SIRT1-ΔExon8 levels returned to basal +48 hrs after stress-stimulation. This differential sensitivity to stress-exposure was confirmed in vivo in mouse tissues (Figures 3C and 3D). Whole-body γ-irradiation of mice consistently induced up-regulation of SIRT1-ΔExon8 expression, while SIRT1-FL expression displayed no change or was moderately down-regulated (Figures 3C and 3D). Note, since SIRT1-ΔExon8 stress-induction consists of a transient pulse (Figures 3A and 3B), tissue-specific kinetics may significantly influence the extent to which SIRT1-ΔExon8 stress-induction is observed in each mouse tissue at the 2.5 hr timepoint used here (Figures 3C and 3D).

SIRT1-FL and SIRT1-ΔExon8 also displayed divergent RNA stability following transcriptional inhibition (Figures 2E and S5). Indeed, the results imply a post-transcriptional switch which stimulates increased SIRT1-ΔExon8 production from a pre-existing pool of immature SIRT1 pre-mRNA during transcriptional inhibition. The dynamics of RNA processing are complex, however our preliminary analyses indicate that protein levels of the SR-family splicing factor SC35 are down-regulated by stress-exposure (Figure 2F), while selective SC35 depletion also coincides with SIRT1-ΔExon8 induction (Figure 2F). These results suggest a role for SC35 in regulating the stress-sensitive splicing of SIRT1, consistent with a previous report in mice [34], while SR-family splicing factors are known targets of multiple stress-signaling kinases [35].

### Characterization of SIRT1-ΔExon8 protein expression

**Figure 4:**
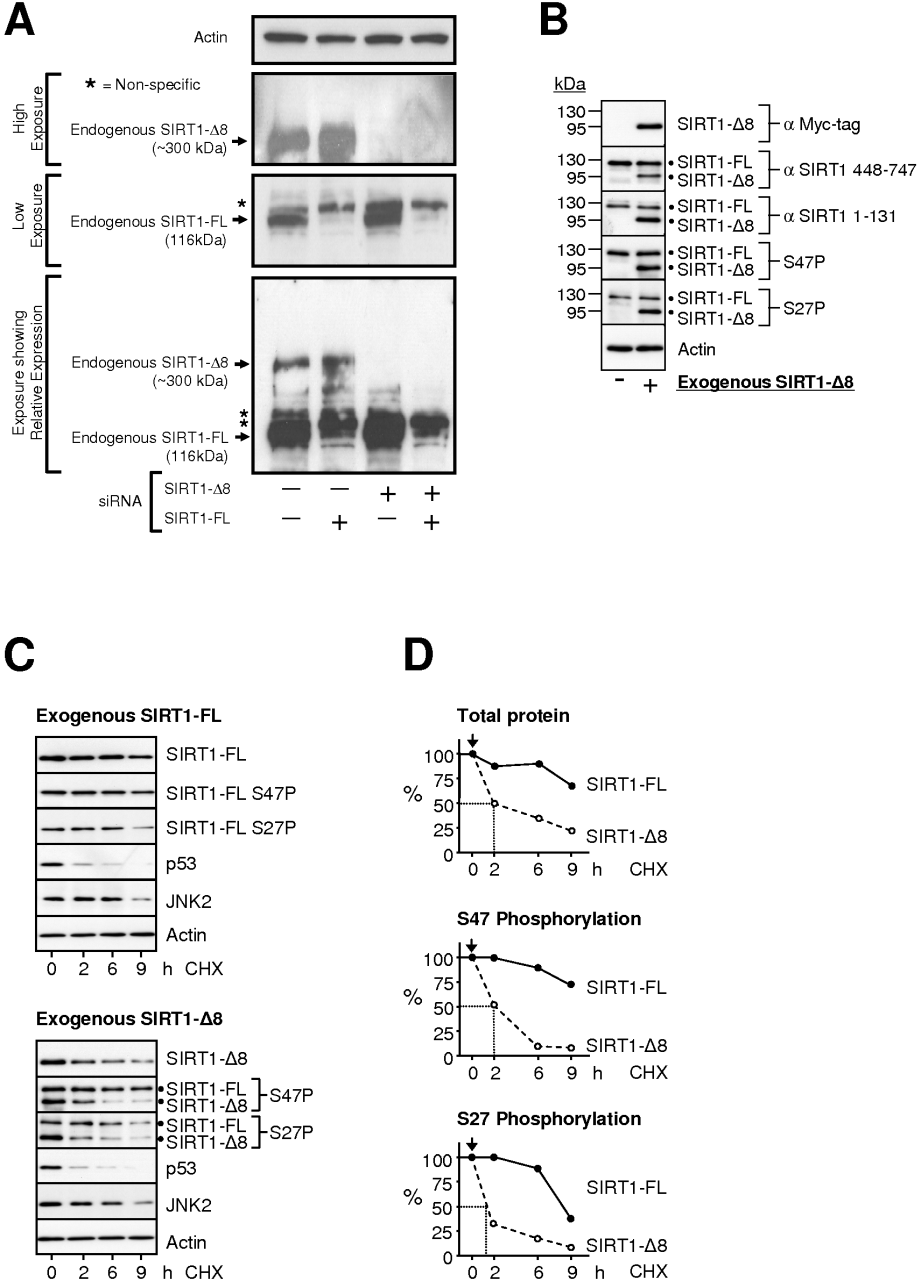
Similarities and differences in SIRT1-ΔExon8 versus SIRT1-FL proteins. (**A**) Validation of endogenous SIRT1-ΔExon8 protein expression. Mouse P19 EC cells were transfected with the indicated siRNAs. 48 hours later, cells were analysed by Western blot for SIRT1 expression in whole cell lysates using a rabbit polyclonal anti-SIRT1 N-terminal specific antibody (SIRT1 residues 1-131), with β-Actin as a loading control. Different exposures are shown to aide visualisation of SIRT1-FL and SIRT1-ΔExon8 proteins and to highlight their relative expression. (**B**) SIRT1-ΔExon8 can be expressed as a protein and phosphorylated at S27 and S47. Myc-tagged SIRT1-ΔExon8 was transiently expressed in HCT116 cells and total cell lysates were probed with an array of antibodies. The C-terminal Myc-tag was probed with anti-c-Myc antibody (top panel), and detects SIRT1-ΔExon8 at ∼95 kDa. Lower panels were probed with SIRT1 antibodies and therefore detect endogenous SIRT1-FL (∼116 kDa, upper band) and exogenous SIRT1-ΔExon8 (∼95 kDa, lower band). Lysates were also probed using a C-terminal-specific SIRT1 antibody (residues 448–747), a N-terminal-specific SIRT1 antibody (residues 1–131), and phospho-specific antibodies for phosphorylated SIRT1-serine27 (S27P) or phosphorylated SIRT1-serine47 (S47P). (**C**) SIRT1-ΔExon8 protein has a short half life compared to SIRT1-FL. Western blots monitor decreases in protein level at intervals following treatment of HCT116 cells with cycloheximide (CHX) to halt protein synthesis. SIRT1-FL and SIRT1-ΔExon8 were probed with using either the myc-tag or anti-SIRT1 antibodies as in (B) above. Levels of p53, JNK2 and Actin were probed in the same samples to provide internal controls displaying their expected protein turnover. (**D**) Graphs summarize the Western blot data (from C, above) by densitometry for levels of total SIRT1 protein, or phosphorylation at S27 or S47. Data is expressed with respect to the highest signal in the corresponding immuno-blots in (C). In each case the half-life is indicated.

Human SIRT1-ΔExon8 cDNA was cloned and engineered into a mammalian expression vector containing a CMV promoter and C-terminal Myc and His tags (Figure S6; Methods). In addition, we designed human and mouse isoform-specific siRNA against SIRT1-ΔExon8 or SIRT1-FL (Figures 1A and 2A) and validated their isoform selectivity at the mRNA level (Figure S7) and protein level (see below). Human SIRT1-FL typically displays an apparent molecular weight of ∼116 kDa by SDS-PAGE. Exogenous expression of SIRT1-ΔExon8 in human cells gave the expected ∼95 kDa SIRT1-ΔExon8 monomeric protein product detectable with antibodies directed against the SIRT1 N-terminus (residues 1–131) (Figure 4B), and against the SIRT1 C-terminus (residues 448–747) (Figure 4B). The 95 kDa SIRT1-ΔExon8 exogenous protein was also detectable with an antibody directed against the C-terminal Myc tag (Figure 4B, top panel). These results confirm that human SIRT1-ΔExon8 is translated in-frame in vivo.

Importantly a 300 kDa Western blot band was also observed following exogenous expression of SIRT1-ΔExon8 in addition to the expected ‘monomeric’ 95 kDa species described above (data not shown). We suggest that the SIRT1-ΔExon8 protein may exist within a large multi-molecular complex which is resistant to the denaturing conditions of SDS-PAGE. Partial exposure of SIRT1-ΔExon8 protein, otherwise buried within a high molecular weight complex, would explain our observation that antibodies directed against discrete SIRT1 domains display differential immuno-affinity towards endogenous SIRT1-ΔExon8 protein at 300 kDa (data not shown). Murine P19 EC cells were found to express high SIRT1-ΔExon8 RNA levels (Figures 2C-2E), and selective siRNA-mediated knockdown of endogenous SIRT1-ΔExon8 resulted in loss of a ∼300 kDa protein band in these cells (Figure 4A). Similarly, endogenous SIRT1-ΔExon8 protein also migrated at 300 kDa in murine fibrosarcoma cells (Figure S8). Future studies will explore this high molecular weight SIRT1-ΔExon8 protein complex in detail. For the present study, we focused on the expected 95 kDa monomeric SIRT1-ΔExon8 protein in human cells (see below). SIRT1-FL is constitutively phosphorylated at serines 27 and 47 in human cells (S27P and S47P)[Refs 36, 37]. Interestingly, SIRT1-ΔExon8 was also phosphorylated at S27 and S47 (Figure 4B), indicating that native SIRT1-kinase(s) recognise and phosphorylate the exogenously expressed SIRT1-ΔExon8 protein.

SIRT1-FL can undergo nucleo-cytoplasmic translocation in a CRM1-dependent manner due to its N-terminal nuclear localization and nuclear export sequences [38]. Cell fractionation experiments demonstrated that SIRT1-FL and SIRT1-ΔExon8 proteins display broadly similar sub-cellular localisation (Figure S9). This suggests that the nuclear-shuttling machinery distributes the two SIRT1 isoforms in a comparable manner. Taken together with the phosphorylation of both SIRT1 isoforms at S27 and S47 (Figure 4B), the results collectively suggest that SIRT1-FL and SIRT1-ΔExon8 present equivalent N-termini in vivo, despite the C-terminal deletion of 186 residues in SIRT1-ΔExon8.

### SIRT1-FL and SIRT1-ΔExon8 display distinct protein half-lives

Turnover of SIRT1-ΔExon8 and SIRT1-FL was explored using time-course analyses following inhibition of cellular protein synthesis with cycloheximide (CHX; Figure 4C). The results demonstrated a short half-life of ∼2 hours for SIRT1-ΔExon8 protein compared with >9 hours for SIRT1-FL (Figures 4C and 4D). It is worth considering the fact that the SIRT1-ΔExon8 transcript displays striking RNA stability (Figure 3E), and is rapidly induced by stress in a pulse-like manner (Figures 3A and 3B), whereas SIRT1-ΔExon8 protein exhibits rapid turnover (Figures 4C and 4D). This combination may ensure transcript abundance during stress, while tightly regulating the persistence of the protein product, a theme reminiscent of several stress response factors including p53, p63, p73, and c-Jun [14], [39], [40].

### SIRT1-ΔExon8 Protein-Protein Interactions

**Figure 5:**
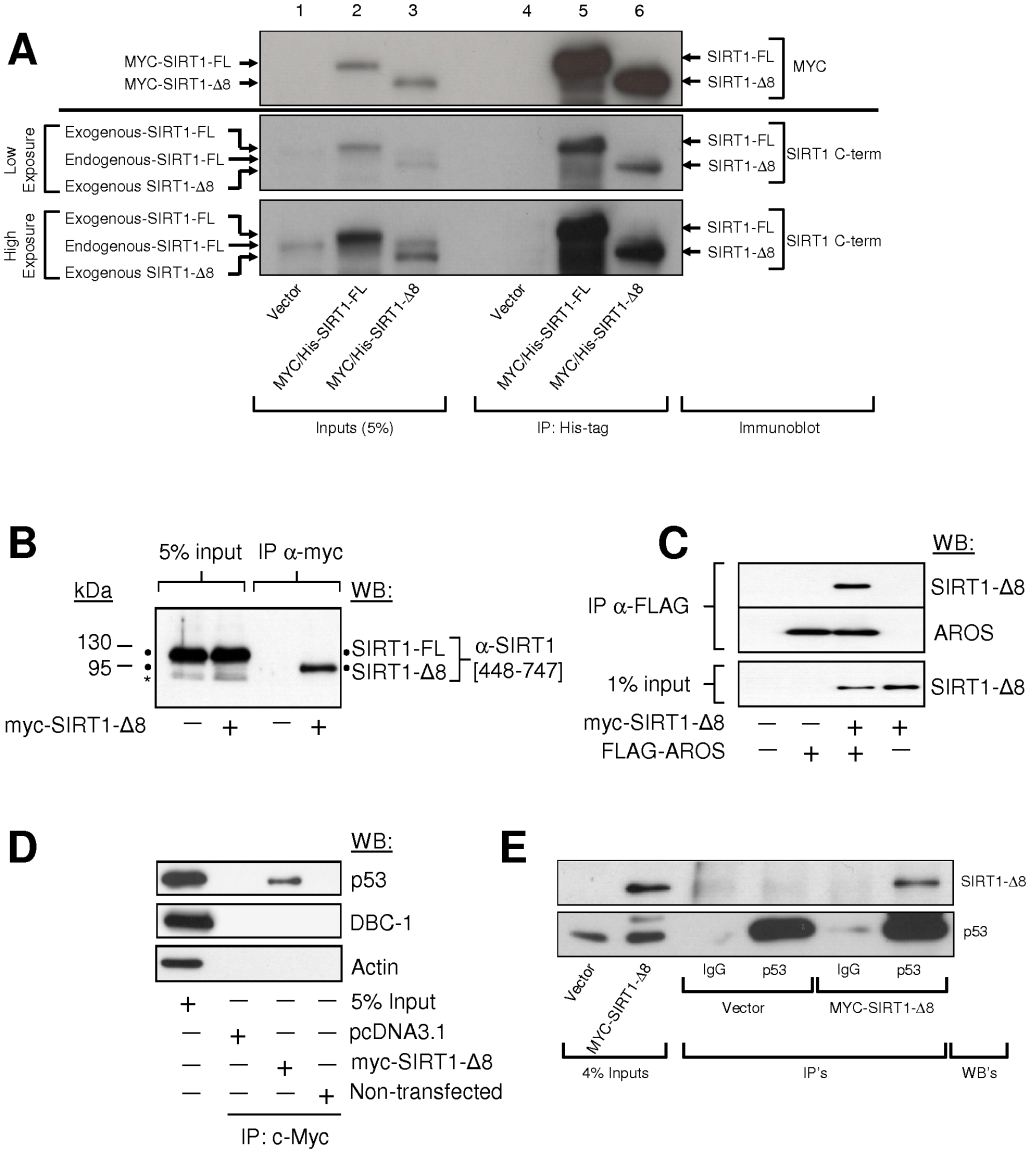
SIRT1-ΔExon8 interacts with AROS and p53, but does not interact with SIRT1-FL or DBC-1. (**A**) SIRT1-ΔExon8 and SIRT FL proteins do not physically interact. Myc/His-tagged SIRT1-ΔExon8, SIRT1-FL or empty vector were exogenously expressed in HCT116 cells followed by affinity purification of the His-tag. Eluates were probed by Western blot for enrichment of Myc-tagged SIRT1-FL and SIRT1-ΔExon8 (upper panel; anti-c-Myc). Lysates were also probed with a C-terminal specific polyclonal SIRT1 antibody (residues 448-747) to visualize endogenous SIRT1-FL in addition to the exogenous SIRT1-FL and SIRT1-ΔExon8 proteins. (**B**) SIRT1-ΔExon8 and SIRT FL proteins do not physically interact. Myc-SIRT1-ΔExon8 was exogenously expressed in HCT116 cells, and cell lysates were immuno-precipitated with anti-c-Myc antibody. Western-blotting was performed with a C-terminal anti-SIRT1 antibody (residues 448–747) as in (A) above. * indicates a non-specific band. (**C**) SIRT1-ΔExon8 interacts with AROS. Myc-SIRT1-ΔExon8 and Flag-AROS were exogenously expressed in HCT116 cells, and the Flag epitope was immuno-precipitated. In the eluate, AROS was detected by probing with an anti-AROS antibody, while the interaction with SIRT1-ΔExon8 was probed with anti-c-Myc antibody. (**D**) SIRT1-ΔExon8 interacts with p53, but not DBC-1. Myc-SIRT1-ΔExon8 was exogenously expressed in HCT116 cells and immuno-precipitated as in (B) above. Eluates were probed for endogenous p53 (anti-p53 DO-1) or endogenous DBC-1 (anti-DBC-1) to assay for interactions with SIRT1-ΔExon8 in vivo. (**E**) SIRT1-ΔExon8 interacts with p53. Myc-SIRT1-ΔExon8 or empty vector were exogenously expressed in HCT116 cells. Whole cell lysates were immuno-precipitated using a polyclonal anti-p53 (FL-393) antibody, followed by Western blotting of eluates for p53 (DO-1, lower panel) or Myc-SIRT1-ΔExon8 with anti-c-Myc antibody.

SIRT1-FL coordinates multiple processes through direct interaction with, and deacetylation of, numerous important regulatory proteins. Therefore we investigated the SIRT1-ΔExon8 protein interactome to deduce its biological role. Purified endogenous SIRT1-FL is reported to form homo-trimers in vivo [41], a characteristic which may be conserved between Sir2-family members [42]. However SIRT1-FL does not interact with SIRT1-ΔExon8 (Figure 5A, lower panel, lane6; and Figure 5B). This indicates a requirement for the Exon8-encoded region to mediate SIRT1-FL-trimerization in vivo. SIRT1-FL deacetylation activity is fine-tuned by several mechanisms [43], including interaction with the SIRT1-FL activator AROS (Active Regulator Of SIRT1–FL)[44], and the SIRT1-FL-inhibitor DBC-1 (Deleted in Breast Cancer-1)[45], [46]. Our immunoprecipitation experiments demonstrated that AROS can also bind to SIRT1-ΔExon8 in vivo (Figure 5C), in agreement with the reported AROS-binding site (SIRT1-residues 114–217)[44] which is present in both SIRT1-FL and SIRT1-ΔExon8 (Figure 1B). In contrast, DBC-1 does not interact with SIRT1-ΔExon8 (Figure 5D), consistent with the overlap between the DBC-1 binding site (SIRT1-residues 210–500)[45], and the region which is absent from SIRT1-ΔExon8 (SIRT1-residues 452–638; Figure 1B). By deduction, SIRT1-residues 452–500 may be critical for the SIRT1-FL-DBC-1 interaction, and/or SIRT1-Exon8-skipping may result in structural changes which hinder a SIRT1-ΔExon8-DBC-1 interaction. In summary, (i) SIRT1-ΔExon8 does not engage in hetero-trimerization with SIRT1-FL; (ii) SIRT1-ΔExon8 can bind AROS (a SIRT1-FL activator); (iii) SIRT1-ΔExon8 does not bind DBC-1 (a SIRT1-FL inhibitor).

Thus, the inclusion/exclusion of Exon8 generates significantly different capacity for SIRT1-protein interactions, raising the possibility of other distinct interactions and functions for SIRT1-ΔExon8 versus SIRT1-FL. In particular, AROS and DBC-1 have been shown to control the ability of SIRT1-FL to complex with, and repress, the crucial tumour suppressor p53 [31], [32], [44]–[46]. Importantly, SIRT1-ΔExon8 also interacted with p53 in vivo (Figures 5D and 5E), indicating that Exon8-exclusion does not interrupt the SIRT1-p53 interaction. This is consistent with the retention on SIRT1-ΔExon8 of 13 of the 14 critical conserved residues required for the interaction between a p53 peptide and a SIRT1-homologue (Figure 1B)[47]. Indeed, it is interesting that the Exon8-encoded region lies immediately downstream of the reported SIRT1-p53 interacting residues (Figure 1B)[47], such that a distal region of the SIRT1 C-terminus becomes more proximal to the interaction site in a p53-SIRT1-ΔExon8 complex (Figure 1B).

### SIRT1-ΔExon8 regulates p53 acetylation

**Figure 6:**
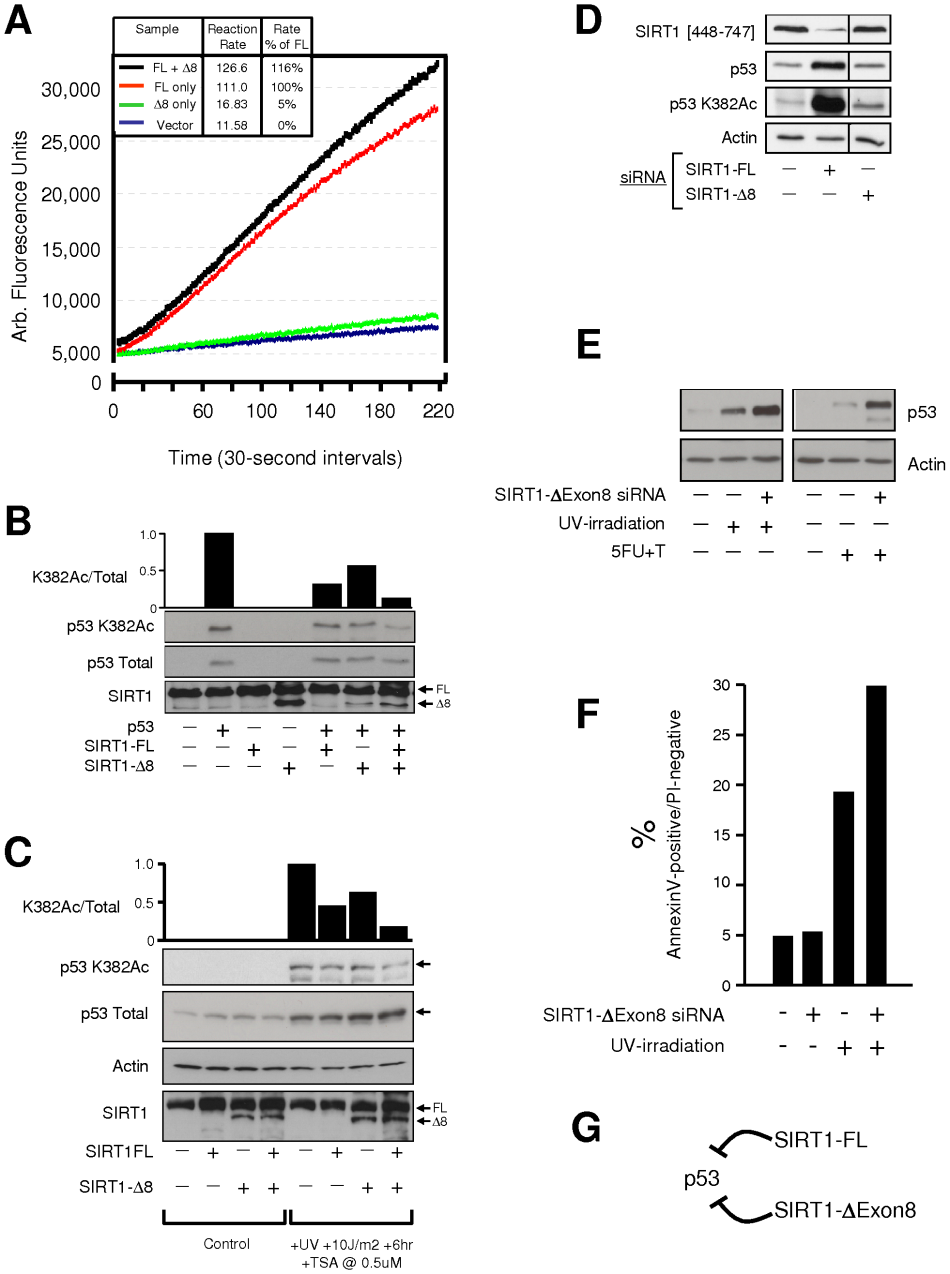
SIRT1-ΔExon8 represses p53acetylation, p53 accumulation and the cellular response to stress. (**A**) SIRT1-ΔExon8 displays weak deacetylase activity in vitro and is additive with SIRT1-FL. SIRT1-ΔExon8, SIRT1-FL or empty vector were exogenously expressed in HCT116 cells followed by affinity purification as in Figure 5A above. In the fluorometric deacetylase assay, the initial reaction rate for each condition was calculated (Methods) and expressed as a percentage of the rate obtained for purified SIRT1-FL alone. (**B**) Analysis of SIRT1-ΔExon8 deacetylase activity by exogenous expression in vivo. Human HCT116 p53−/− cells were transfected as indicated and whole cell lysates were probed for SIRT1 expression with a rabbit polyclonal anti-SIRT1 N-terminal specific antibody (residues 1–131). Total levels of p53 and p53 acetylation at Lysine 382 (p53 K382Ac) were also probed. The relative intensity of p53 K382 acetylation was quantified and corrected by the total amount of p53 present using densitometry. (**C**) Analysis of SIRT1-ΔExon8 deacetylase activity following cell stress in vivo. Human HCT116 p53+/+ cells were transfected as indicated and whole cell lysates were analysed by Western blotting for SIRT1, total p53, and p53 K382Ac levels exactly as in (B) above, in addition to densitometry to quantify the relative intensity of p53 K382 acetylation. (**D**) Selective silencing of SIRT1-FL or SIRT1-ΔExon8 has distinct effects on p53 acetylation. HCT116 cells were transfected with SIRT1-isoform-specific siRNA to specifically knockdown SIRT1-FL or SIRT1-ΔExon8 (Methods). Total cell lysates were probed for endogenous SIRT1-FL (anti-SIRT1 C-terminal residues 448-747), for endogenous p53 (anti-p53 DO-1), and for p53 acetylation at K382 (p53 K382Ac). β-Actin was probed as a loading control. (**E**) Depletion of SIRT1-ΔExon8 levels correlates with enhancement of p53-accumulation after stress. Western blots for p53 and β-Actin are shown in human ARPE19 cells which were treated as indicated and harvested 24 hrs after stress exposure (for experimental overview, see Figure S12). (**F**) Depletion of SIRT1-ΔExon8 levels correlates with enhanced apoptosis after stress. Cells were treated exactly as in (E) above. Following cell harvest, Annexin V-staining and FACS analysis were performed to quantify the percentage of cells passing through the early stages of apoptosis. (**G**) Schematic summary of this figure: both SIRT1-FL and SIRT1-ΔExon8 can repress p53.

SIRT1-FL is an NAD-dependent deacetylase of p53 [31], [32]. The deacetylase potential of SIRT1-ΔExon8 was assessed using a standard fluorometric in vitro deacetylase assay (see: Methods). In this assay, the target substrate is a p53 peptide acetylated at Lysine382, which is a well-documented substrate for SIRT1-FL-mediated deacetylation [31], [32]. As expected, SIRT1-FL displayed robust deacetylase activity in vitro (Figure 6A). In contrast, SIRT1-ΔExon8 displayed weak but measurable deacetylase activity (Figures 6A and S11). Importantly, the presence of SIRT1-FL and SIRT1-ΔExon8 in a 1∶1 ratio (Figure S10, lane 4) was not inhibitory, instead combining to produce an additive effect on deacetylation of the p53 peptide, with deacetylase activity increased ∼15% above that of SIRT1-FL alone (Figure 6A). The deacetylase activity of SIRT1-ΔExon8 was also assessed in vivo. Exogenous SIRT1-FL displayed greater deacetylation of exogenous p53 at K382 than SIRT1-ΔExon8; however exogenous expression of both SIRT1-FL plus SIRT1-ΔExon8 resulted in enhanced deacetylation of p53 in vivo (Figure 6B). Similar results were observed with endogenous p53 following stress-exposure (Figure 6C). Conversely, selective siRNA-mediated depletion of endogenous SIRT1-FL or SIRT1-ΔExon8 caused increased p53 K382 acetylation, again consistent with a strong deacetylation role for SIRT1-FL and weak deacetylation by SIRT1-ΔExon8 (Figure 6D). In summary, in vivo and in vitro deacetylase results indicated that: (i) directly or indirectly both SIRT1 isoforms were capable of down-regulating p53 K382 acetylation; (ii) SIRT1-FL exerted a stronger effect on p53 K382 acetylation than SIRT1-ΔExon8; (iii) in combination, the two SIRT1 isoforms exerted an additive effect.

### SIRT1-ΔExon8 regulates p53 expression and the cellular stress response

Since both p53 and SIRT1-ΔExon8 are strongly induced by stress, we asked if SIRT1-ΔExon8 might regulate p53 following acute DNA damage (UV-irradiation) or chronic disturbance of RNA metabolism (5-FU plus Thymidine). Stress-induced up-regulation of p53 expression levels is well-documented and was observed here as expected (Figure 6E)[16, 22; reviewed in: 13, 14]. However, depletion of SIRT1-ΔExon8 significantly enhanced stress-induced up-regulation of p53 expression levels (Figure 6E), indicating a repressive role for SIRT1-ΔExon8 in p53 regulation after stress. Next, given the central role of p53 in directing the cellular response to stress, and the ability of SIRT1-ΔExon8 to repress p53 levels, we also examined the possibility that SIRT1-ΔExon8 might influence the cellular response to stress. Depletion of SIRT1-ΔExon8 using siRNA prior to exposure of cells to three different stresses resulted in a markedly more severe stress phenotype at the whole-cell level (Figure S12). This effect was quantified using FACS analysis and Annexin V staining which indicated a significantly increased fraction of cells passing through early stages of apoptosis if SIRT1-ΔExon8 was depleted prior to stress-exposure, compared to stress-exposure alone (Figure 6F). Taken together, the results suggest an anti-apoptotic role for SIRT1-ΔExon8 following stress (Figures 6F and S12), consistent with a repressive role for SIRT1-ΔExon8 in p53 regulation after stress (Figures 6E and 6G).

### p53 regulates SIRT1 splicing to complete an auto-regulatory loop

We reasoned that a decision must be made between synthesizing SIRT1-FL or SIRT1-ΔExon8 from the primary SIRT1 pre-mRNA transcript, and that this decision must be coordinated with the crucial requirements of the p53 stress response. Thus, we sought to understand the regulation of SIRT1 splicing.

**Figure 7:**
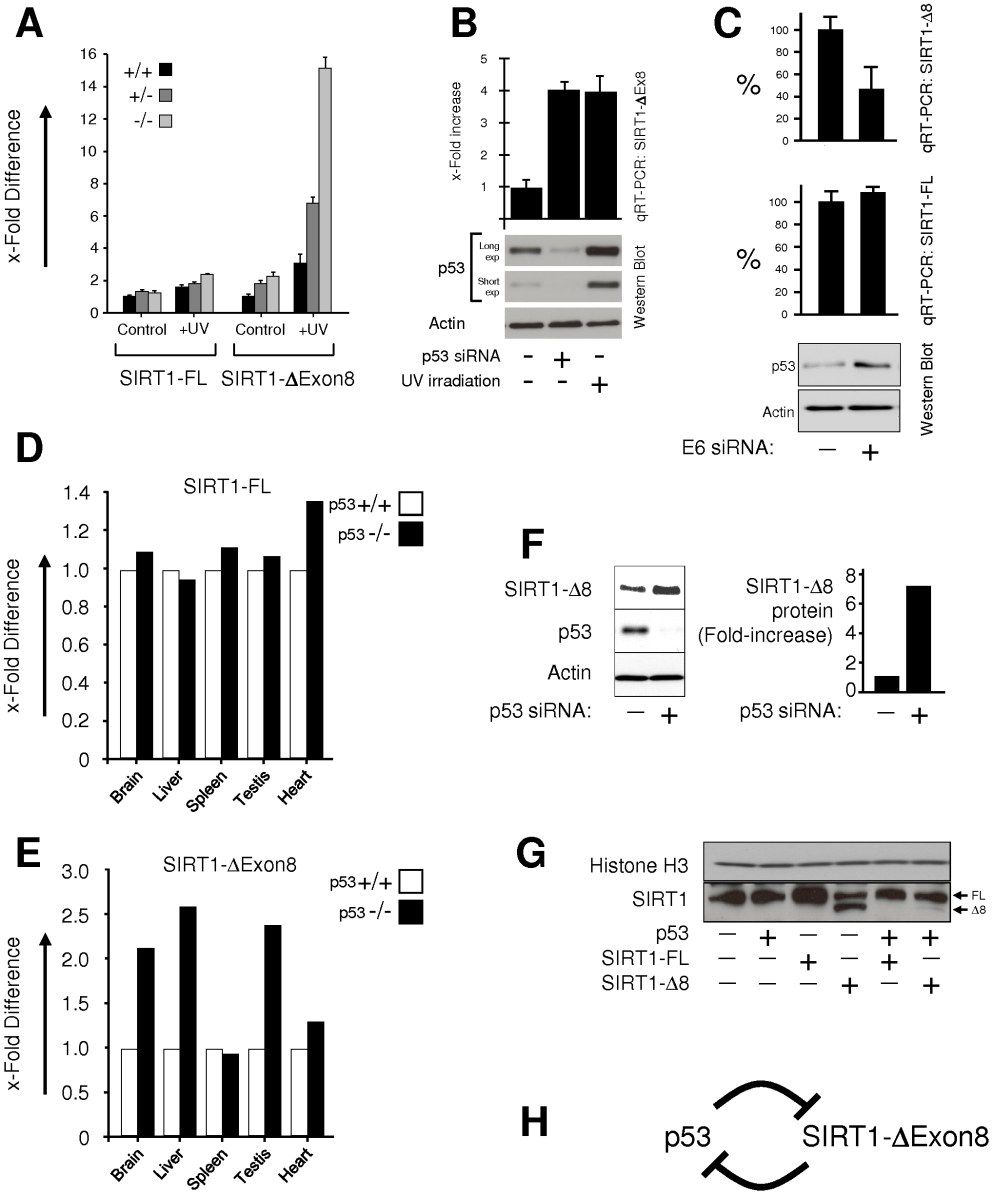
p53 represses SIRT1-ΔExon8, completing a p53-SIRT1-ΔExon8 auto-regulatory feedback loop. (**A**) SIRT1-FL and SIRT1-ΔExon8 mRNA expression by qRT-PCR in HCT116 p53 knockout isogenic clones +24 hrs after mock or UV stress treatment (with 10 J/m^2^), using with polyA-tailed mRNA as template. Similar results were obtained using total RNA as template (data not shown). (**B**) The p53-SIRT1-ΔExon8 axis was analysed in ARPE19 normal human epithelial cells following treatments as indicated (UV-irradiation with 10J/m^2^), followed by analysis of SIRT1-ΔExon8 mRNA levels by qRT-PCR and Western blotting for total p53, with β-Actin as loading control. (**C**) Abrogation of HPV-16 E6-mediated repression of p53 levels, results in down-regulation of SIRT1-ΔExon8 mRNA levels. Lower panel: HPV-16-infected human cervical epithelial SiHa cells were transfected with siRNA to deplete the HPV-protein E6, resulting in de-repression of p53 protein expression. β-Actin used as loading control. SIRT1-FL and SIRT1-ΔExon8 mRNA levels were also measured by qRT-PCR. Increased p53 expression following E6-depletion correlated with selective down-regulation of SIRT1-ΔExon8 mRNA levels. (**D**) SIRT1-FL mRNA levels are independent of p53 expression in vivo. Mouse SIRT1-FL mRNA expression was analysed by qRT-PCR and expressed relative to β-Actin by the delta-C(t) method in a panel of mouse tissues from wt p53+/+ mice and p53−/− littermates. Data is shown with respect to levels in p53+/+ mice for each tissue. (**E**) SIRT1-ΔExon8 mRNA levels are dependent on p53 expression in vivo. Mouse SIRT1-ΔExon8 mRNA expression was analysed by qRT-PCR and expressed relative to β-Actin in tissues from wt p53+/+ mice and p53−/− littermates exactly as in (D) above. (**F**) SIRT1-ΔExon8 protein levels are repressed by p53 in vivo. ARPE19 normal human epithelial cells were transfected with control or p53 siRNA. 48 hours later, the cells were transfected with SIRT1-ΔExon8 and the protein levels of SIRT1-ΔExon8, p53 and β-Actin were analysed by Western blot (left panels). The increase in SIRT1-ΔExon8 protein levels associated with p53 depletion was quantified by densitometry (right panels). (**G**) SIRT1-ΔExon8 protein levels are repressed by p53 in vivo. Human HCT116 p53−/− cells were transfected as indicated and whole cell lysates were probed 24 hours later for SIRT1 expression with a rabbit polyclonal anti-SIRT1 N-terminal specific antibody (residues 1–131). Total Histone H3 levels were also probed as a loading control. (**H**) A schematic which summaries the reciprocal relationship between p53 and SIRT1-ΔExon8. A p53-SIRT1-ΔExon8 auto-regulatory feedback loop is observed, whereby p53 can represses SIRT1-ΔExon8 expression (Figures 7A–7G) and SIRT1-ΔExon8 can repress p53 (Figures 6A–6E).

In human cell experiments using p53 gene knockout (Figure 7A), siRNA-mediated silencing of p53 expression (Figure 7B), or p53 de-repression by depletion of the p53-repressor E6 (Figure 7C), SIRT1 splicing clearly displayed p53-dependency. In particular, SIRT1-ΔExon8 mRNA expression was selectively attenuated by p53 before and after stress, in contrast to SIRT1-FL mRNA levels (Figures 7A–7C). This ability of p53 to selectively repress SIRT1-ΔExon8 mRNA expression exhibited conservation between humans and mice, with increased SIRT1-ΔExon8 mRNA expression levels evident in the brain, liver and testis of p53−/− mice compared to their wt p53+/+ control littermates (Figures 7D and 7E). However, we must point out that comparing other tissues of p53+/+ and p53−/− mice, the ability of p53 to repress SIRT1-ΔExon8 mRNA expression displayed some tissue specificity, with no effect of p53 knockout on SIRT1-ΔExon8 levels evident in mouse spleen and heart tissues (Figures 7D and 7E). We next investigated whether p53 affected SIRT1-ΔExon8 protein levels in addition to SIRT1-ΔExon8 mRNA expression. Depletion of p53 via siRNA was associated with increased SIRT1-ΔExon8 protein expression (Figure 7F). Conversely, exogenous SIRT1-ΔExon8 protein expression was significantly higher in a p53-null background than when co-expressed with exogenous wt p53 protein (Figure 7G; see also, above: Figure 6B, lower panel). In summary, p53 exerted a repressive effect on expression levels of the SIRT1-ΔExon8 protein, consistent with p53-dependent repression of SIRT1-ΔExon8 mRNA levels (see above). Therefore, since p53 can repress SIRT1-ΔExon8 (Figure 7), while in reciprocal SIRT1-ΔExon8 can repress p53 acetylation and expression (Figure 6), the results collectively suggest a p53-SIRT1-ΔExon8 feedback loop whereby p53 auto-regulates its expression levels via p53-dependent SIRT1 splice variation (Figure 7H).

## Discussion

Here we characterize the first alternative SIRT1 isoform. In mammals, multiple copies of the sequence motif UAGG in any single Exon are associated with skipping of that Exon by alternative splicing [49], [50]. In SIRT1, the only coding Exon which contains the UAGG motif is SIRT1-Exon8 (human Exon8  = 4 copies; mouse Exon8  = 4 copies), consistent with evolutionary conservation of SIRT1 Exon8 skipping, as shown here in human and mouse SIRT1.

SIRT1-FL protein has a predicted molecular weight of 81 kDa, however it is observed at 116 kDa by SDS-PAGE analysis. Accordingly, monomeric SIRT1-ΔExon8 protein is predicted to migrate at ∼95 kDa by SDS-PAGE. We detected SIRT1-ΔExon8 protein at 95 kDa and 300 kDa in human epithelial cells (Figure 4B and data not shown), while it migrated at 300 kDa in mouse fibroblast and embryonic lineages (Figures 4A and S8). This may be explained by the possible involvement of SIRT1-ΔExon8 in detergent-resistant higher molecular weight complexes which exhibit species-specific and/or tissue-specific differences. Consistent with this, both Sir2p (the yeast homologue of SIRT1) and SIRT6 (a mammalian Sirtuin family member) are known to participate in large multi-protein complexes [51], [52], while tissue-specific SIRT1 functions have also been reported [53], [54]. Additional uncharacterized SIRT1 bands have also been observed by Western blot in brain, thymus, lung and testis tissues [54]–[56], consistent with the tissues where we observed higher levels of SIRT1-ΔExon8 expression (Figures 1D and 2B); however, whether these bands represent SIRT1-ΔExon8 or indeed arise by alternative splicing remains unclear at this point. Taken together with our report, the emerging evidence suggests that the ability of SIRT1 to regulate mammalian biology at several levels may be partially explained by the existence of alternate SIRT1 isoforms, such as SIRT1-ΔExon8.

p53 expression levels and activity dictate p53-dependent stress-responses, tumour suppression, and whether p53 is pro- or anti-ageing, while SIRT1 is implicated in the regulation of p53, ageing and the stress response (see: Introduction). It is therefore notable that the newly discovered splice variant SIRT1-ΔExon8 is stress-responsive (Figure 3), p53-dependent (Figure 7) and can regulate p53 in reciprocal (Figure 6). This reveals novel auto-regulatory feedback between p53 and SIRT1, key factors in the common biology of stress, cancer, metabolism, and ageing. Importantly, the kinetics of SIRT1-ΔExon8 stress-induction (Figures 3A and 3B) overlap with its ability to regulate p53 acetylation, p53 accumulation and the cellular stress response (Figure 6). Stress-induction of SIRT1-ΔExon8 is also consistent with the numerous stress-responsive roles which have been demonstrated previously for SIRT1 in promoting DNA repair and stress tolerance [8], [57], [58]. Similarly, caloric restriction (CR) is reported to induce SIRT1 expression, for example in kidney cells [59]. Therefore it would be interesting to examine the effect of CR on SIRT1-ΔExon8 expression in kidney, as this tissue exhibited the largest stress-induction of SIRT1-ΔExon8 in mice (Figure 3D). Significant potential exists for differential regulation of the p53 stress response by SIRT1-FL and SIRT1-ΔExon8 given that: (i) SIRT1-ΔExon8 is stress-responsive but SIRT1-FL is not (Figures 3A-3D); (ii) SIRT1-ΔExon8 displays differential RNA stability and protein stability versus SIRT1-FL (Figures 3E, 4C and 4D); (iii) SIRT1-ΔExon8 and SIRT1-FL also display differential capacity to interact with AROS (a SIRT1-activator which promotes p53-deacetylation)[44] or DBC-1 (a SIRT1-inhibitor which promotes p53-acetylation)[45], [46](Figures 5C and 5D). More generally, it is likely that SIRT1-FL and SIRT1-ΔExon8 perform distinct cellular roles since phosphorylation has emerged as an important regulator of SIRT1-FL function [37], [43], [60]. Thus, divergent regulation of the two SIRT1 isoforms may arise via differential phosphorylation of SIRT1-FL versus SIRT1-ΔExon8, since the Exon8-encoded region contains several novel phosphorylated residues which are substrates for stress-signaling kinases and a cyclinB/cdk1 docking site [60]. In contrast, the stress-activated CK2 kinase was shown to dictate SIRT1-FL deacetylation activity, substrate-affinity, and SIRT1-FL-dependent cellular stress-tolerance through phosphorylation at Ser 154, 649, 651 and 683, all of which are retained on SIRT1-ΔExon8 [61].

Although SIRT1-ΔExon8 displays partial deletion of the deacetylase domain, it retains some deacetylation activity (Figure 6). The deacetylase experiments used here employ p53 acetylated at Lys382 as a well-defined target substrate. However in vivo, SIRT1-FL has numerous other target substrates, to which SIRT1-ΔExon8 may have differential affinity or may deacetylate to a greater or lesser extent. Similarly, in vivo conditions will differ from the in vitro deacetylase assay, for example, substrate and NAD concentrations are in excess in vitro. Propionylation and butyrylation of lysine residues are highly novel protein post-translational modifications with as-yet unknown purposes, but which are chemically similar to acetylation [62]. In addition to deacetylation, SIRT1-FL was recently reported to also perform de-propionylation and de-butyrylation of C-terminal Lysines on p53 [63] and Histone H3 Lys23 [64], although currently, it remains unclear how SIRT1-FL differentiates between these reactions. While the internal deletion of SIRT1-ΔExon8 attenuates its deacetylation of p53, the possibility remains that it may favour a role for SIRT1-ΔExon8 in other processes such as de-propionylation or de-butyrylation. On a different note, SIRT1-FL has been reported to possess deacetylation-independent functions, such as inhibition of NFkB activity [65], stimulation of the methyltransferase SUV39H1 [66], and repression of the epithelial Na^+^-channel alpha sub-unit [67]. Interestingly, these deacetylation-independent functions require only the SIRT1 N-terminus [65]-[67], a region which remains intact in SIRT1-ΔExon8. Similarly, SIRT1-dependent neuroprotection against low potassium conditions can also be conferred by deacetylase-dead SIRT1-FL mutant proteins [68]. Taken together, these reports raise the possibility that SIRT1-ΔExon8 could influence p53 and whole-cell behaviour despite its weak deacetylase activity via deacetylase-independent functions.

The mechanism underlying p53-dependent SIRT1 splicing remains unclear. However, p53 can bind to single-stranded RNA with ∼100-fold higher affinity than double-stranded DNA in vitro [69]. Moreover, several studies describe direct RNA binding by p53 suggesting a general role in RNA regulation, for example inhibition of RNA helicases, modification of RNA secondary structures, and catalysis of RNA-annealing [70]-[73]. So, although p53 has been primarily characterised as a DNA-binding transcription factor, p53 also possesses the capacity to interact directly with RNA and may therefore be in a position to directly regulate SIRT1 RNA splicing. Alternatively, given our results indicating a role for the splicing factor SC35 in the regulation of SIRT1-ΔExon8 splicing (Figure 3F), we are also currently exploring the possibility that p53 might influence RNA processing through modulation of the SR-family of splicing factors.

p53 and SIRT1 both influence development, for example during embryonal stages and in neuronal tissues [6], [8], [16]–[18], where RNA splicing also has a central role [34], [74]. In addition, p53 and SIRT1 influence metabolic processes, such as respiration efficiency, glucose and lipid metabolism, and metabolic shifts during dietary restriction [1]–[5], [21]. Therefore the existence of an auto-regulatory loop involving p53-dependent modulation of SIRT1 alternative splicing may provide insight into the coordination of these complex processes. Significant debate currently exists regarding whether SIRT1 is a tumour-promoter or a tumour-suppressor, and whether SIRT1 can mediate lifespan extension via caloric restriction in higher organisms. Our discovery of alternate SIRT1 isoforms with distinct characteristics provides important insight into SIRT1 function and may provide an explanation for previous apparently conflicting results. Similarly, numerous other SIRT1-attributed functions (see: Introduction) may in fact be distributed between SIRT1-FL and SIRT1-ΔExon8, working independently or in synergy. Novel therapeutics targeting specific SIRT1 isoforms may enable tailored manipulation of p53 or SIRT1 functions in a wide variety of disease states.

## Materials and Methods

### Ethics Statement

Mice were treated in accordance with the Spanish Laws and the Guidelines for Humane Endpoints for Animals Used in Biomedical Research. The Spanish National Cancer Research Centre (CNIO) is part of the “Carlos III” Health Institute (ISCIII) and all protocols were previously subjected and approved by the Ethical Committee of the ISCIII; approval ID numbers: PA-45 v2, PA-312 and PA-130/07.

### Mouse Experiments

p53+/+ and p53-/- mice of pure C57BL6 genetic background [75] were housed in a pathogen-free barrier area under standard conditions. 14-week-old mice were exposed to 5 Gy of whole-body γ-irradiation and sacrificed +2.5 hours after stress-exposure for immediate tissue harvest.

#### Cell culture and treatments

p53+/+, p53+/− and p53−/− isogenic clones derived from human HCT116 colorectal epithelial cancer cells [76] were a gift from Dr. Bert Vogelstein (John Hopkins University). ARPE19 normal human non-transformed retinal epithelial cells [77] were purchased from ATCC. All cells used in this study were cultured according to the corresponding ATCC guidelines: WI-38, HT-29, HT-1080, LoVo, RKO, MCF-7, TOV112D, SW48, SW480, SW620, DLD-1, U2OS, SAOS-2, SiHa, wt MEFs, ARF KO MEFs, mouse P19 Embryonal Carcinoma cells. Mouse JGA 95.2 Fibrosarcoma cells were derived in the Tumour Suppression Laboratory, CNIO, Spain. Cells were treated with 200 nM Actinomycin D (Sigma, A9415) to inhibit transcription (Figures 3E and S5), as described [78]. UV-irradiation was performed as described [79], delivering 0 (control), 5, 10 or 20 J/m^2^ of UV-irradiation as indicated in each Figure. 5-FU was used at 375 µM and Thymidine was used at 425 µM (Figures 6E and S12). Protein half-life experiments using 100 µg/mL cycloheximide (Figures 4C and 4D) as described [37]. Cells passing through the early stages of apoptosis were quantified by fluorescence-activated cell sorting (FACS) using Annexin V-Fluos (Roche) as described [80].

#### Transfection and siRNA sequences

Transfection of siRNA (all from Dharmacon) and/or DNA expression constructs were carried out as described [80], [81]. Sequences and target loci of all human and mouse siRNA are detailed in Figure S13.

#### RNA, RT-PCR and primer sequences

Total RNA and oligo-dT-purified polyA-tailed mRNA were isolated as described [22]. Normal human tissue total RNA samples were purchased from AMS Biotechnology (Europe). Normal mouse tissue total RNA samples were prepared as follows: whole tissues were snap-frozen in liquid nitrogen immediately after mouse sacrifice. Tissue total RNA was extracted by the standard Trizol method (Invitrogen #15596-026) using a polytron homogenizer, followed by phenol-chloroform separation and iso-propyl alcohol precipitation. RT-PCR and real-time quantitative RT-PCR (qRT-PCR) were performed for 30–45 cycles as described [22]. All human qRT-PCR data in this study represent the mean +/− standard deviation of three determinations. Also, expression of SIRT1-FL and SIRT1-ΔExon8 mRNA levels was calculated relative to the housekeeper β-Actin using the delta–C(t) method in mouse tissues and cell lines. The loci of all human primers used for SIRT1 RT-PCR in this study are depicted in Figure 1A (human primer sequences are listed in Figure S14). Human SIRT1 splice-variant-specific primers were as follows: SIRT1-FL –Forward: Ex7/8F; Reverse: Ex8R. For specific PCR amplification of SIRT1-ΔExon8 -Forward: Ex6F; Reverse: Ex7/9R. The primers used for co-amplification of SIRT1-FL plus SIRT1-ΔExon8 in Figure 1C and Figures S1, S4 and S5, were: Forward: Ex7F; Reverse: Ex9R. Equivalent RNA loading was routinely assessed in RT-PCR samples by monitoring GAPDH and LaminA/C housekeeper mRNA levels with primers as described [81].

The loci of all mouse primers used for SIRT1 RT-PCR in this study are depicted in Figure 2A (mouse primer sequences are listed in Figure S15). Mouse SIRT1 splice-variant-specific primers were as follows: SIRT1-FL –Forward: Ex7/8F; Reverse: Ex8R. For specific PCR amplification of SIRT1-ΔExon8 -Forward: Ex6F; Reverse: Ex7/9R. For co-amplification of SIRT1-FL plus SIRT1-ΔExon8 in Figure S3, primers were: Forward: Ex7F; Reverse: Ex9R. We also monitored total SIRT1 mRNA levels (data not shown) using the primer pair: Forward: Ex1F; Reverse: Ex2R (see Figure 2A). PCR products were visualised on a UV transilluminator using 1.2% Agarose gel electrophoresis and 200 ng/mL ethidium bromide (Sigma, A9415). Sequencing was performed by MWG Biotech.

#### Expression Constructs

pcDNA3 mammalian expression vector encoding Flag-tagged human AROS [44] was kindly provided by Eun-Joo Kim and Soo-Jong Um, Sejong University, Seoul, Korea. Cloning of SIRT1-FL and SIRT1-ΔExon8: from HCT116 p53−/− cells was performed in a two step process due to the high GC-content of Exon1 (Cloning strategy, Figure S6). In the first step, the first four Exons were amplified by RT-PCR and cloned in TOPO cloning vector, pCR2.1 (Invitrogen) using forward primer SIRT1-BamF1 with BamHI linker (5′-GTCGAGCGGGAGCAGAGGAGGC-3′) and the reverse primer SIRT1-R5 (5′-CATCGCTTGAGGATCTGGAAG-3′). A BamHI/EcoRV fragment from a pCR2.1 plasmid was shuttled to pBSK+. The remaining parts of SIRT1-FL and SIRT1-ΔExon8 without stop codons were amplified by using forward primer SEx2 (5′-GTGAAAGTGATGAGGAGGATAG-3′) and reverse primer SIRT1-rRV with EcoRV linker (5′-GCGATATCTGATTTGTTTGATGGATAGTTCATG-3′) and joined in the BglII/EcoRV-cut pBSK+ plasmid. Both SIRT1-FL and SIRT1-ΔExon8 were then shuttled across pcDNA3.1MycHis plasmid (Invitrogen).

Total cell lysate protein preparation, SDS-PAGE, and immuno-blotting: as described [80], [82], except Cell Signaling antibodies, where supplier's protocol was followed precisely. The antibodies used in this study are listed in Figure S16. Cell fractionation was performed using nuclear and cytoplasmic extraction reagents (NE-PER kit, Thermo Scientific). Western blotting visualisation by ECL (Roche). For densitometry, Western blot protein band intensity was quantified and corrected by the corresponding β-Actin or total p53 internal control using “Quantity One v.4.5” image analysis software (Biorad).

Immunoprecipitations: for Myc-tagged SIRT1 constructs (Figures 5B and 5D), Mammalian c-Myc Tag IP/Co-IP kit from Pierce (#23625) was used. Immunoprecipitation of FLAG-AROS (Figure 5C) used FLAG-tagged protein immunoprecipitation kit (Sigma, FLAGIPT1), with elution using FLAG-peptide. Immunoprecipitation for Figure 5E was as described [80] using ProteinG-Sepharose Fast Flow beads (Sigma #P-3296).

In vitro deacetylase assay: was performed on SIRT1-FL or SIRT1-ΔExon8 proteins which had been expressed in, and purified from, human HCT116 cells. The assay was performed according to the manufacturer's protocol (Abnova #KA0105), including no-enzyme, no NAD and no-substrate controls (data not shown). The reaction rate was calculated from the slope of the linear regression best-fit line to each curve during the linear phase of the reaction for each condition in the fluorometric deacetylase assay.

## Supporting Information

Figure S1 An additional SIRT1 transcript is revealed by RT-PCR with multiple primer pairs. PCR primers, with target loci as indicated in Figure 1A, were used in pairs to co-amplify SIRT1-FL and SIRT1-ΔExon8 transcripts from total RNA from human HCT116 cells. PCR products were analysed by agarose gel electrophoresis. The expected band sizes for amplification from SIRT1-FL and the observed band sizes are indicated below the panel. In each lane the upper band is the expected SIRT1-FL amplicon, while sequencing of the lower band confirmed a SIRT1 transcript lacking precisely Exon8 only (data not shown). M  =  DNA ladder as marker lane. N  =  Negative controls using no input RNA.
(1.23 MB TIF)

Figure S2 SIRT1-FL versus SIRT1-ΔExon8 expression in a panel of human epithelial cell lines of normal- or cancer-origin. Relative expression of SIRT1 splice variants in a variety of human normal and cancer cell lines. Results show splice-variant-specific qRT-PCR of SIRT1-FL or SIRT1-ΔExon8 (see Methods).
(0.72 MB TIF)

Figure S3 An additional SIRT1 transcript is revealed by RT-PCR with multiple primer pairs in Mouse cells. Mouse PCR primers, with target loci as indicated in Figure 2A, were used in pairs to amplify SIRT1-FL and/or SIRT1-ΔExon8 transcripts from total RNA from MEFs. PCR products were analysed by agarose gel electrophoresis. The expected band sizes are indicated below the panel. The presence of two bands generated with the primer pair in Lane 1 correlates exactly with the expected amplicon from SIRT1-FL (upper band), and a SIRT1 transcript lacking precisely Exon8 only (lower band). The other lanes demonstrate splice variant specific RT-PCR for SIRT1-FL or SIRT1-ΔExon8. M  =  DNA ladder as marker lane. N  =  Negative controls using no input RNA.
(1.43 MB TIF)

Figure S4 The kinetics of SIRT1-ΔExon8 stress-induction are dose-dependent. RT-PCR co-amplification of SIRT1-FL and SIRT1-ΔExon8 (two bands, see: Methods) reveals the rapid kinetics of SIRT1-ΔExon8 mRNA induction after UVstress in HCT116 cells. Also, higher stress insult correlates with greater SIRT1-ΔExon8 induction, significantly altering the relative abundance of the two SIRT1 transcripts. The results correlate with the specific qRT-PCR of SIRT1-FL or SIRT1-ΔExon8 shown in Figure 3A.
(1.48 MB TIF)

Figure S5 SIRT1-FL versus SIRT1-ΔExon8: relative abundance and mRNA stability. HCT116 cells were incubated in the presence of the transcriptional inhibitor Actinomycin D (see: Methods) and total RNA was harvested at intervals for RT-PCR analysis. SIRT1-FL and SIRT1-ΔExon8 were co-amplified (see: Methods) to monitor changes in their relative abundance. The results correlate with the specific qRT-PCR of SIRT1-FL or SIRT1-ΔExon8 shown in Figure 3E: following transcriptional inhibition, levels of SIRT1-FL mRNA decay rapidly whereas SIRT1-ΔExon8 levels are increased.
(1.48 MB TIF)

Figure S6 Cloning and expression of SIRT1-FL and SIRT1-ΔExon8. Strategy of cloning SIRT1 and SIRT1-Δ8 in mammalian expression plasmid. Both SIRT1 and SIRT1-Δ8 were amplified from HCT116 p53-/- cells by RT-PCR in a two step process (see: Methods).
(0.66 MB TIF)

Figure S7 Selective silencing via siRNA. Splice-variant-specific depletion of human SIRT1-FL or SIRT1-ΔExon8 was achieved using the siRNA shown in Figure 1A. HCT116 cells were transfected with siRNA targeting human SIRT1-FL or SIRT1-ΔExon8 (see: Methods). Transcript levels of SIRT1-FL (lower panel) or SIRT1-ΔExon8 (upper panel) were measured by qRT-PCR and corrected for loading using the mRNA levels of the housekeeper GAPDH. Data  =  Mean +/- Std Deviation of 3 determinations.
(0.65 MB TIF)

Figure S8 Detection of endogenous SIRT1-ΔExon8 protein in mouse fibrsarcoma cells. Mouse Fibrosarcoma cells were transfected with the siRNA indicated. Whole cell lysates were prepared as described in Methods, analysed by SDS-PAGE and blotted using an anti-SIRT1 (1-131) N-terminalspecific antibody. Blots were also probed for β-Actin as an internal loading control.
(1.46 MB TIF)

Figure S9 Biochemical fractionation to analyse the subcellular localisation of SIRT1-ΔExon8 and SIRT1-FL proteins. Human HCT116 cells were transfected with SIRT1-ΔExon8 or SIRT1-FL and subjected to biochemical fractionation +24 hours afterwards (Methods). Western blotting of each fraction for SIRT1-ΔExon8 or SIRT1-FL was performed using their Myc-tag, with equal cell numbers loaded in each lane. Blotting for Lamin A/C, p53 and Histone H3 was also performed as as internal controls indicating successful fractionation. A moderate difference was discernible in the nuclear soluble fraction between SIRT1-FL and SIRT1-ΔExon8.
(0.96 MB TIF)

Figure S10 Analysis of the expression levels of purified His-SIRT1-FL and His-SIRT1-ΔExon8 for use in the deacetylase assay. HCT116 cells were transfected with the constructs indicated, followed by His-tag immunoprecipitation via Ni-Agarose columns as described in Methods. The levels of SIRT1-FL and SIRT1-ΔExon8 in the eluates were analysed by SDS-PAGE and blotting for the c-MYC tag.
(0.91 MB TIF)

Figure S11 SIRT1-ΔExon8 has weak deacetylase activity in vitro. HCT116 cells were transfected with empty vector or SIRT1-ΔExon8, followed by His-tag immunoprecipitation via Ni-Agarose columns as described in Methods(see also: Supplementary Figure 10). This data is reproduced from Figure 6A, but here only SIRT1-ΔExon8 and the vector-only control are shown. Deacetylase activity of purified SIRT1-ΔExon8 protein was analysed by the fluorometric deacetylase assay and was greater than the background signal in the vector-only control. The table shows the reaction rate as a percentage of the deacetylase activity of SIRT1-FL (see: Figure 6A). The reaction rate was the slope of the linear regression best-fit line to each curve during the linear phase of the reaction.
(0.68 MB TIF)

Figure S12 SIRT1-ΔExon8 displays an anti-apoptotic role after stress. ARPE19 cells were treated with siRNA for 48 hrs, then mock-treated or exposed to stress as indicated. Photographs taken +24 hrs after stress exposure.
(3.82 MB TIF)

Figure S13 siRNA sequences used in this study. All siRNA are written in sense orientation 5′-3′.
(0.72 MB TIF)

Figure S14 Human PCR primer sequences used in this study.
(0.73 MB TIF)

Figure S15 Mouse PCR primer sequences used in this study.
(0.73 MB TIF)

Figure S16 Antibodies used in this study.
(0.79 MB TIF)
