# Supplementary figures and images for: SIRT1 Undergoes Alternative Splicing in a Novel Auto-Regulatory Loop with p53
Source: PLoS One. 2010 Oct 21;5(10):e13502. doi: 10.1371/journal.pone.0013502 (PMC2958826; doi:10.1371/journal.pone.0013502)

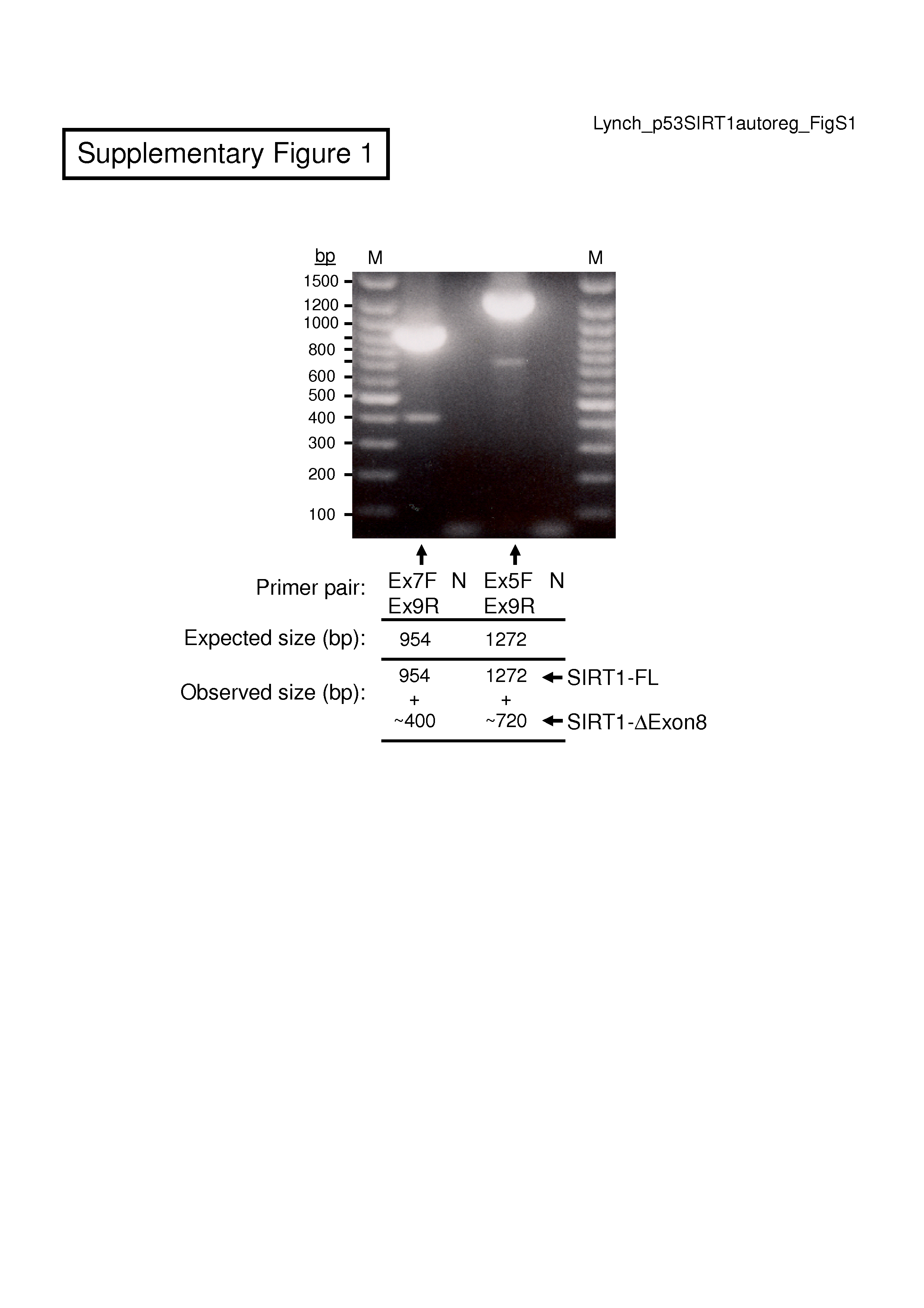

Supplement: Figure S1 — An additional SIRT1 transcript is revealed by RT-PCR with multiple primer pairs. PCR primers, with target loci as indicated in Figure 1A, were used in pairs to co-amplify SIRT1-FL and SIRT1-ΔExon8 transcripts from total RNA from human HCT116 cells. PCR products were analysed by agarose gel electrophoresis. The expected band sizes for amplification from SIRT1-FL and the observed band sizes are indicated below the panel. In each lane the upper band is the expected SIRT1-FL amplicon, while sequencing of the lower band confirmed a SIRT1 transcript lacking precisely Exon8 only (data not shown). M = DNA ladder as marker lane. N = Negative controls using no input RNA. (1.23 MB TIF) [file pone.0013502.s001.tif]

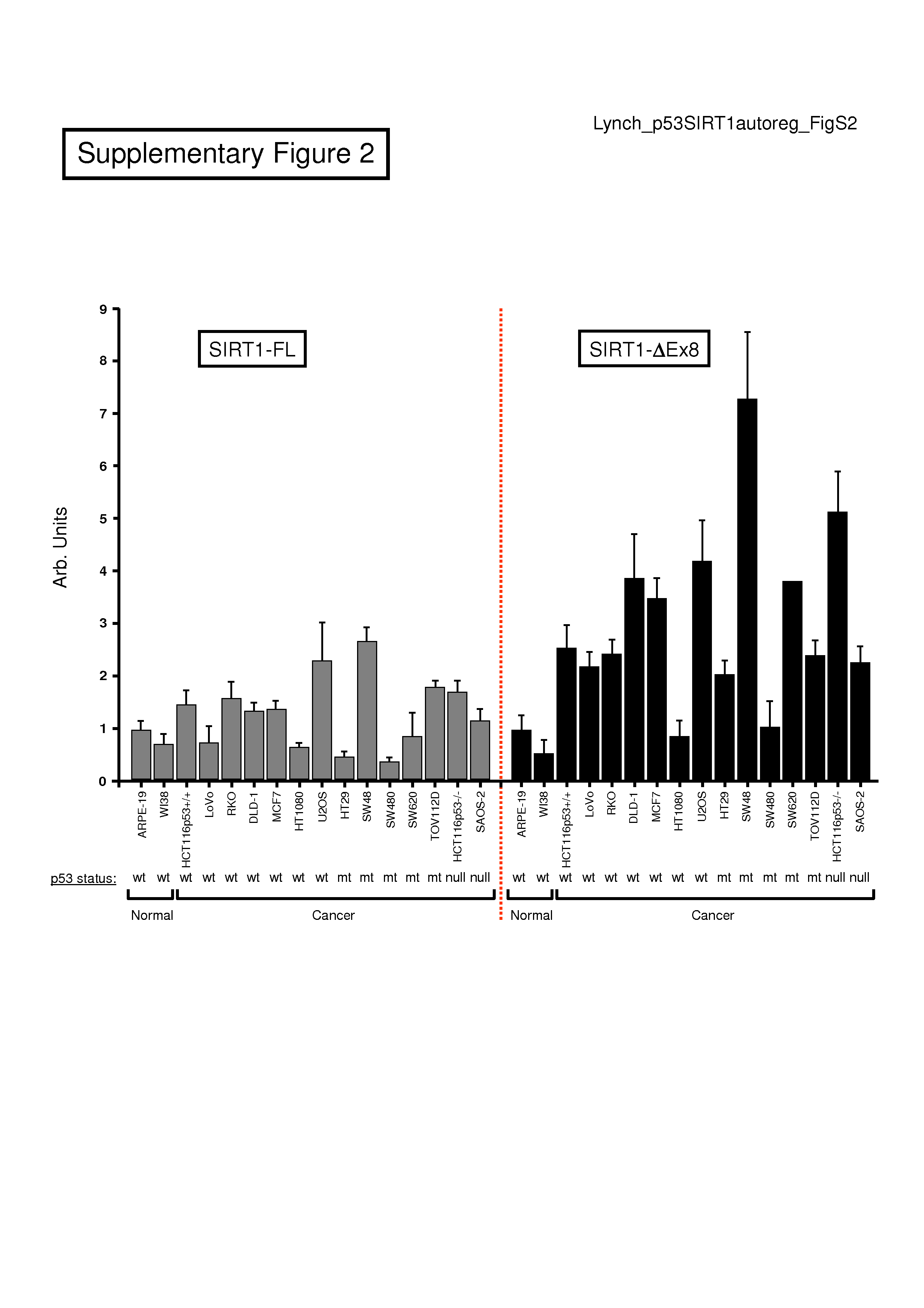

Supplement: Figure S2 — SIRT1-FL versus SIRT1-ΔExon8 expression in a panel of human epithelial cell lines of normal- or cancer-origin. Relative expression of SIRT1 splice variants in a variety of human normal and cancer cell lines. Results show splice-variant-specific qRT-PCR of SIRT1-FL or SIRT1-ΔExon8 (see Methods). (0.72 MB TIF) [file pone.0013502.s002.tif]

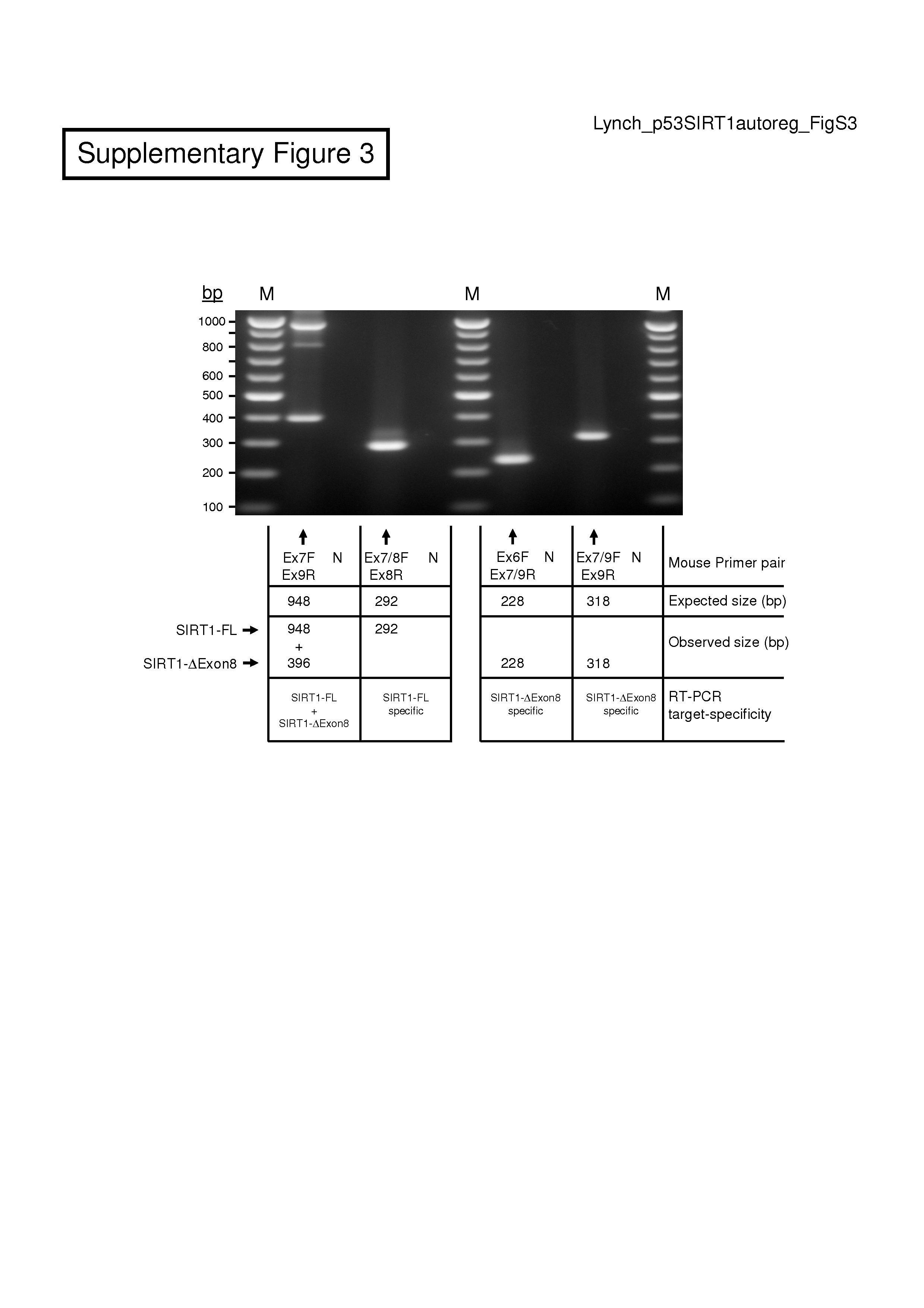

Supplement: Figure S3 — An additional SIRT1 transcript is revealed by RT-PCR with multiple primer pairs in Mouse cells. Mouse PCR primers, with target loci as indicated in Figure 2A, were used in pairs to amplify SIRT1-FL and/or SIRT1-ΔExon8 transcripts from total RNA from MEFs. PCR products were analysed by agarose gel electrophoresis. The expected band sizes are indicated below the panel. The presence of two bands generated with the primer pair in Lane 1 correlates exactly with the expected amplicon from SIRT1-FL (upper band), and a SIRT1 transcript lacking precisely Exon8 only (lower band). The other lanes demonstrate splice variant specific RT-PCR for SIRT1-FL or SIRT1-ΔExon8. M = DNA ladder as marker lane. N = Negative controls using no input RNA. (1.43 MB TIF) [file pone.0013502.s003.tif]

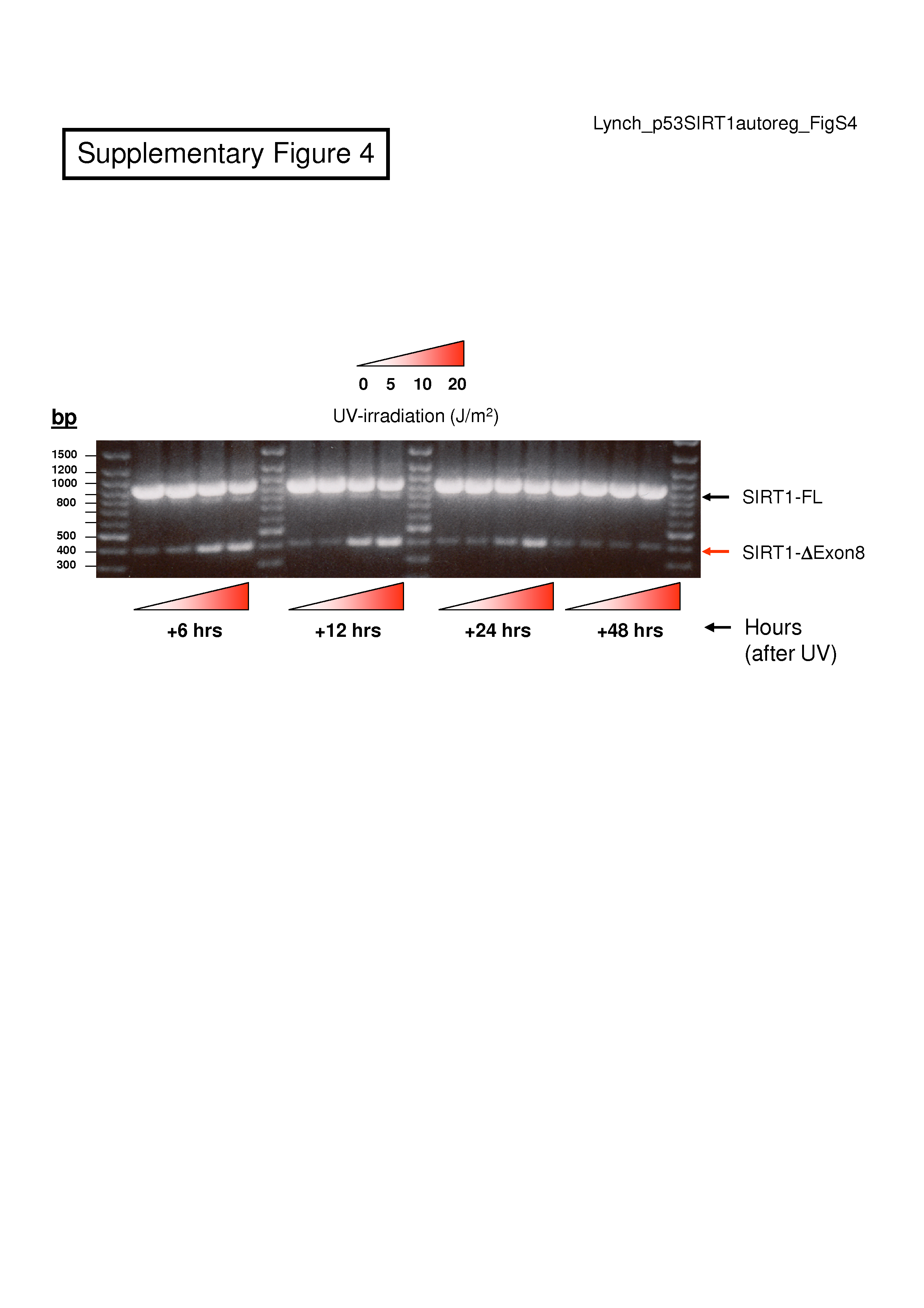

Supplement: Figure S4 — The kinetics of SIRT1-ΔExon8 stress-induction are dose-dependent. RT-PCR co-amplification of SIRT1-FL and SIRT1-ΔExon8 (two bands, see: Methods) reveals the rapid kinetics of SIRT1-ΔExon8 mRNA induction after UVstress in HCT116 cells. Also, higher stress insult correlates with greater SIRT1-ΔExon8 induction, significantly altering the relative abundance of the two SIRT1 transcripts. The results correlate with the specific qRT-PCR of SIRT1-FL or SIRT1-ΔExon8 shown in Figure 3A. (1.48 MB TIF) [file pone.0013502.s004.tif]

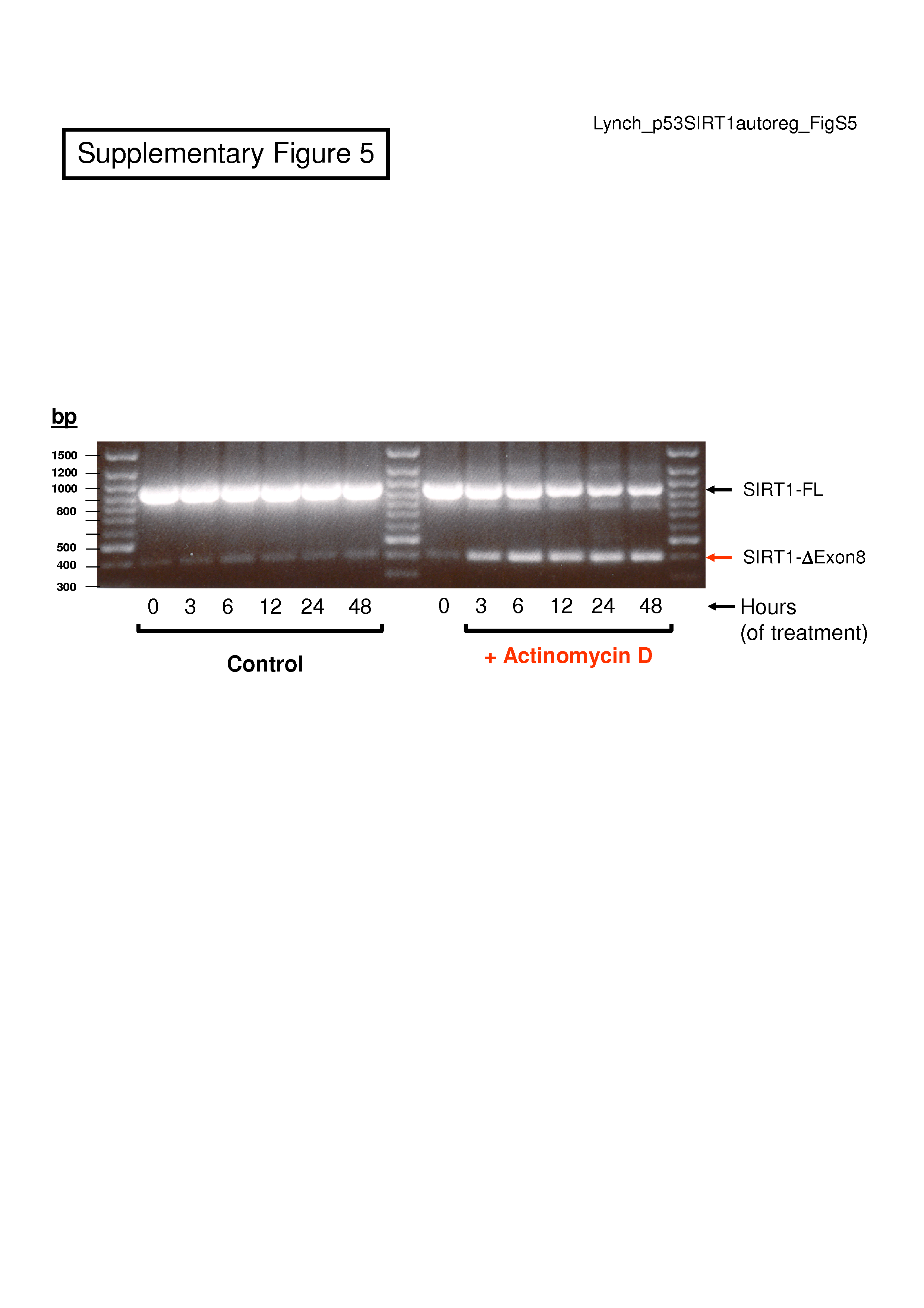

Supplement: Figure S5 — SIRT1-FL versus SIRT1-ΔExon8: relative abundance and mRNA stability. HCT116 cells were incubated in the presence of the transcriptional inhibitor Actinomycin D (see: Methods) and total RNA was harvested at intervals for RT-PCR analysis. SIRT1-FL and SIRT1-ΔExon8 were co-amplified (see: Methods) to monitor changes in their relative abundance. The results correlate with the specific qRT-PCR of SIRT1-FL or SIRT1-ΔExon8 shown in Figure 3E: following transcriptional inhibition, levels of SIRT1-FL mRNA decay rapidly whereas SIRT1-ΔExon8 levels are increased. (1.48 MB TIF) [file pone.0013502.s005.tif]

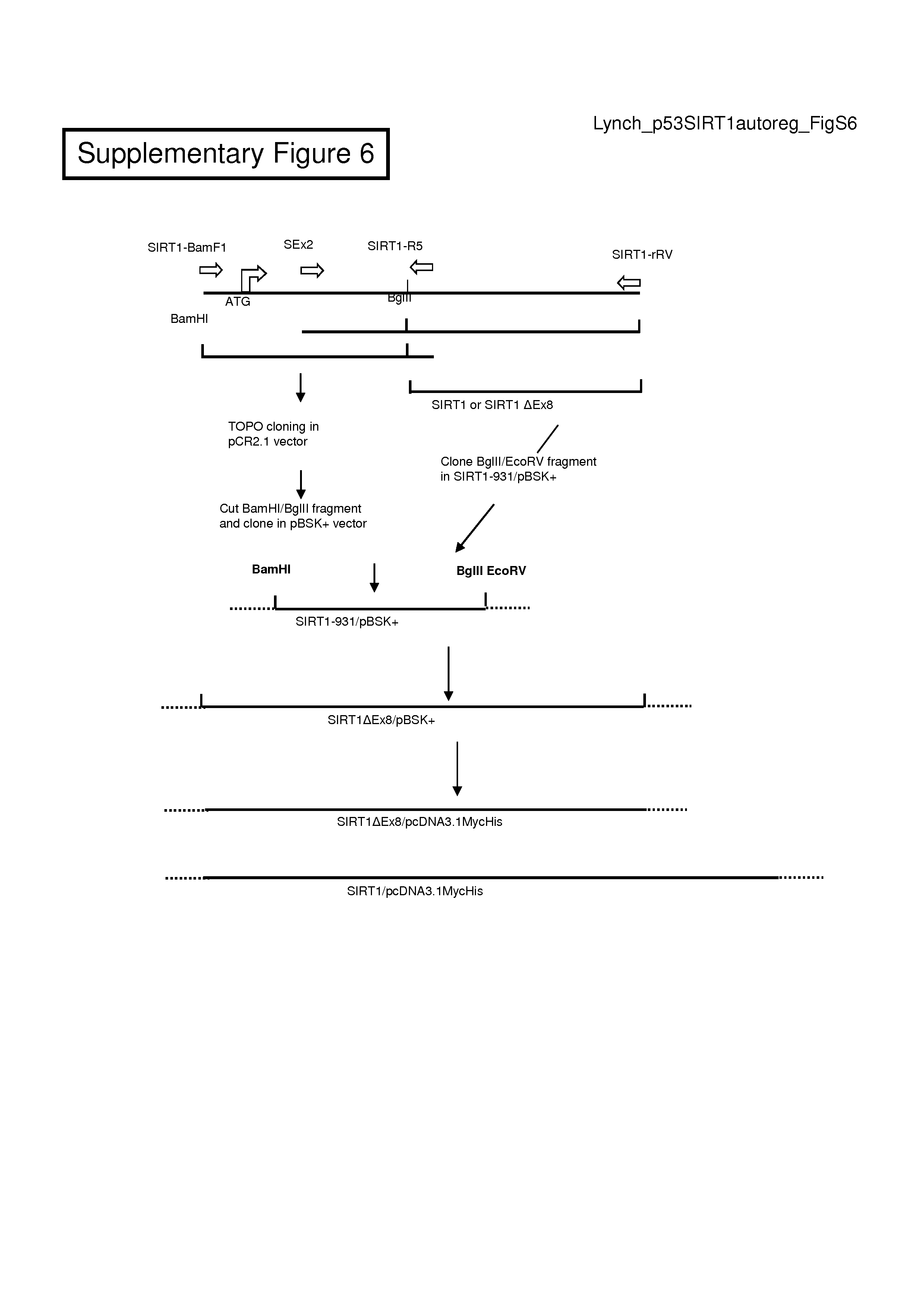

Supplement: Figure S6 — Cloning and expression of SIRT1-FL and SIRT1-ΔExon8. Strategy of cloning SIRT1 and SIRT1-Δ8 in mammalian expression plasmid. Both SIRT1 and SIRT1-Δ8 were amplified from HCT116 p53-/- cells by RT-PCR in a two step process (see: Methods). (0.66 MB TIF) [file pone.0013502.s006.tif]

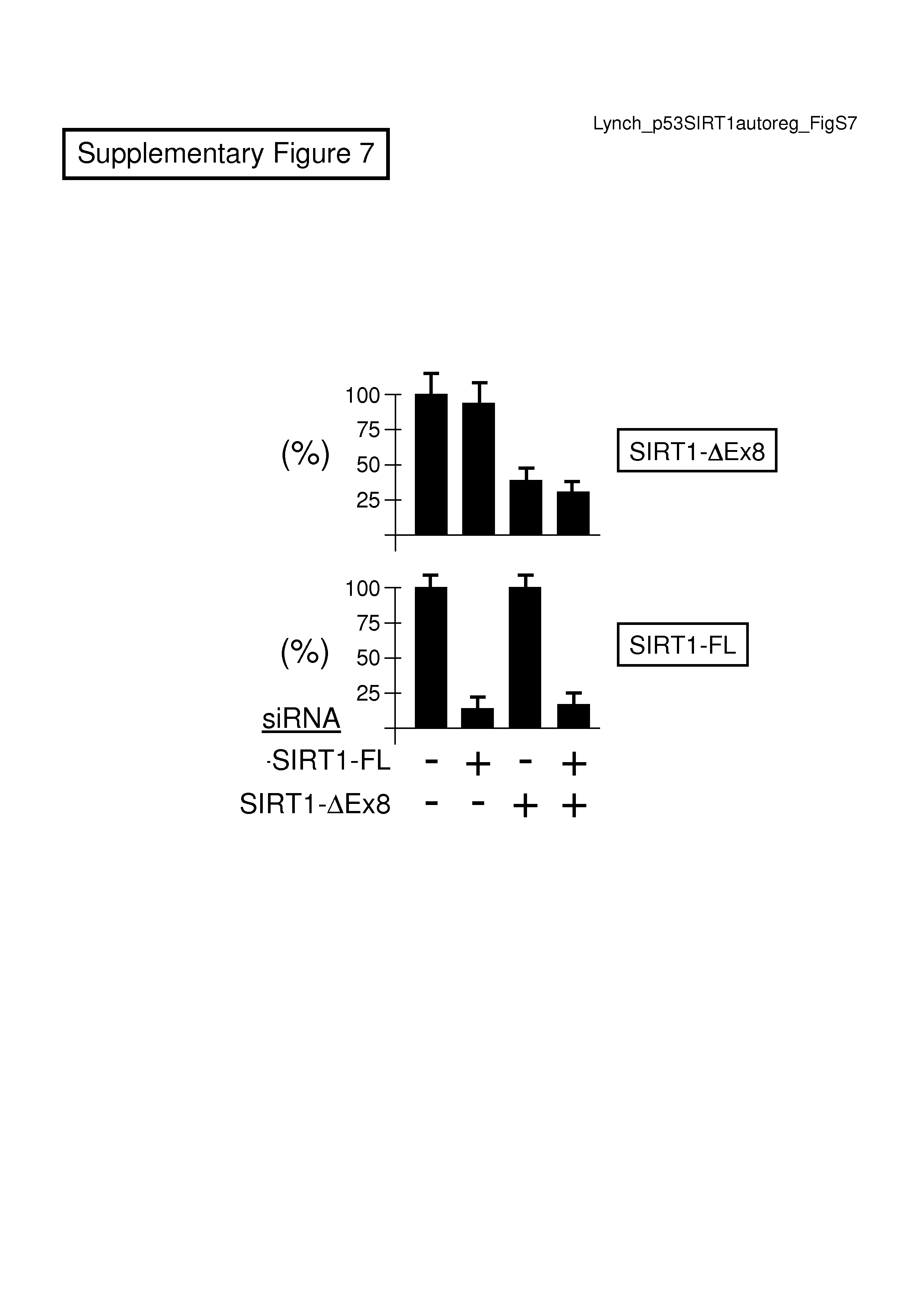

Supplement: Figure S7 — Selective silencing via siRNA. Splice-variant-specific depletion of human SIRT1-FL or SIRT1-ΔExon8 was achieved using the siRNA shown in Figure 1A. HCT116 cells were transfected with siRNA targeting human SIRT1-FL or SIRT1-ΔExon8 (see: Methods). Transcript levels of SIRT1-FL (lower panel) or SIRT1-ΔExon8 (upper panel) were measured by qRT-PCR and corrected for loading using the mRNA levels of the housekeeper GAPDH. Data = Mean +/- Std Deviation of 3 determinations. (0.65 MB TIF) [file pone.0013502.s007.tif]

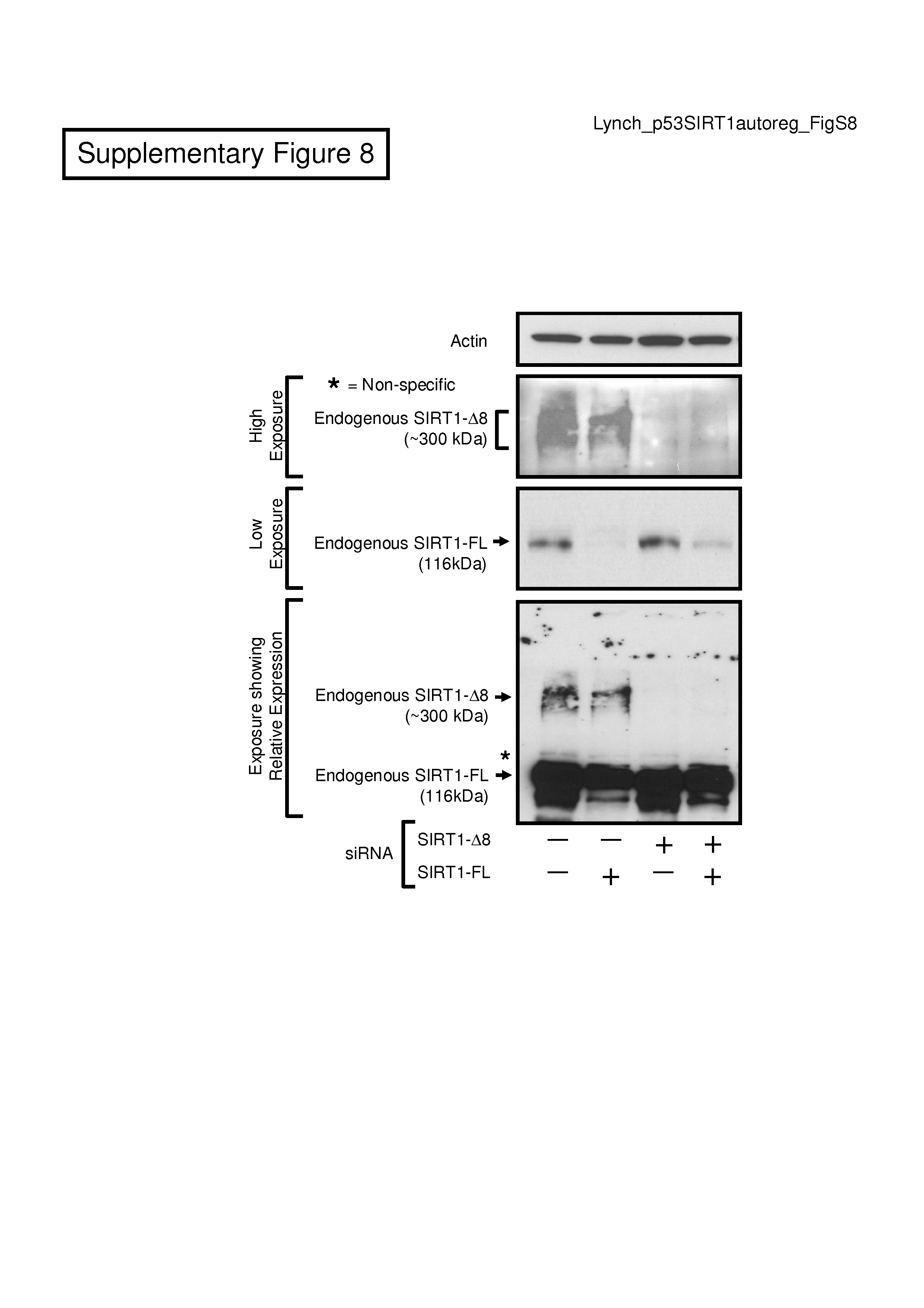

Supplement: Figure S8 — Detection of endogenous SIRT1-ΔExon8 protein in mouse fibrsarcoma cells. Mouse Fibrosarcoma cells were transfected with the siRNA indicated. Whole cell lysates were prepared as described in Methods, analysed by SDS-PAGE and blotted using an anti-SIRT1 (1-131) N-terminalspecific antibody. Blots were also probed for β-Actin as an internal loading control. (1.46 MB TIF) [file pone.0013502.s008.tif]

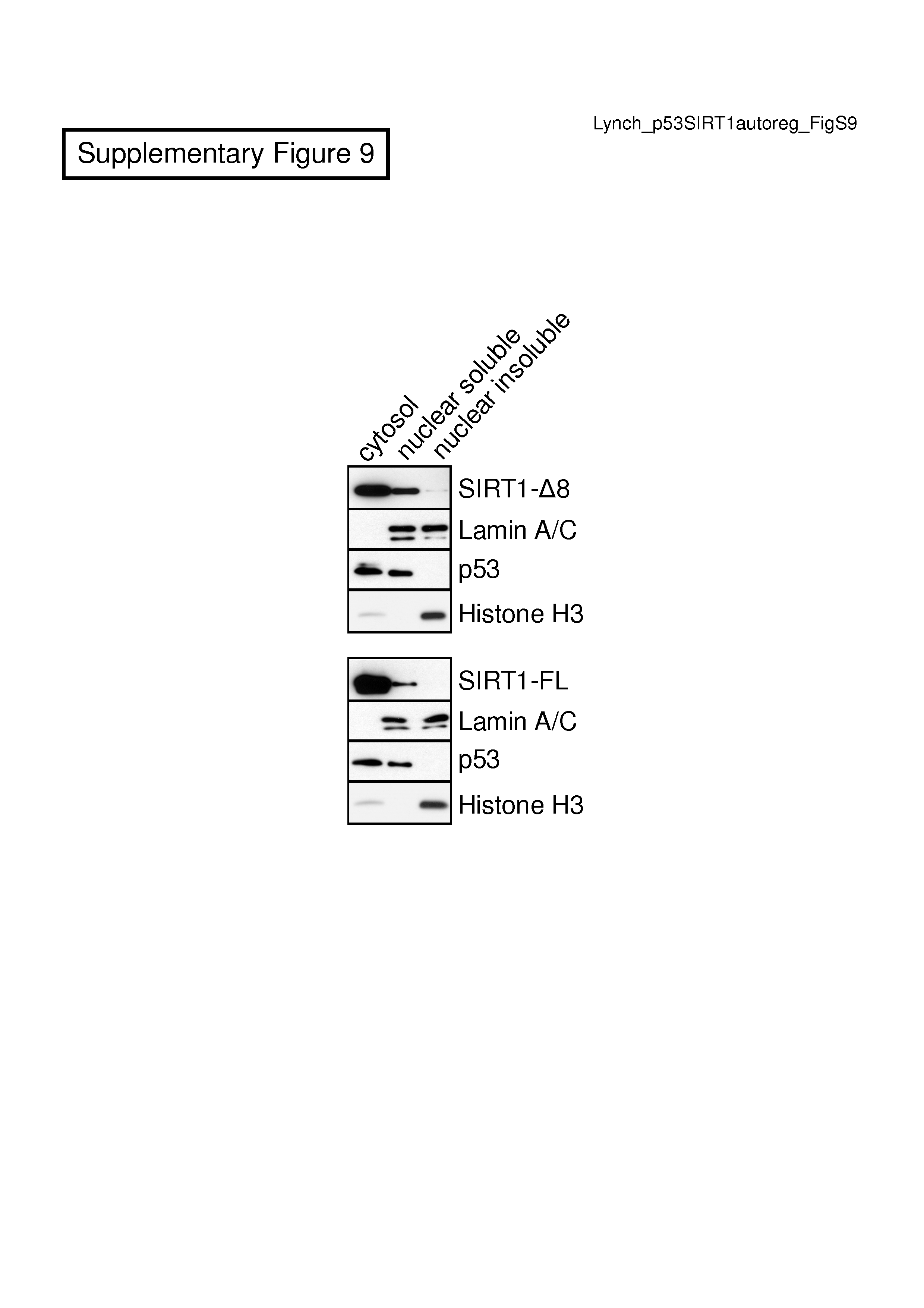

Supplement: Figure S9 — Biochemical fractionation to analyse the subcellular localisation of SIRT1-ΔExon8 and SIRT1-FL proteins. Human HCT116 cells were transfected with SIRT1-ΔExon8 or SIRT1-FL and subjected to biochemical fractionation +24 hours afterwards (Methods). Western blotting of each fraction for SIRT1-ΔExon8 or SIRT1-FL was performed using their Myc-tag, with equal cell numbers loaded in each lane. Blotting for Lamin A/C, p53 and Histone H3 was also performed as as internal controls indicating successful fractionation. A moderate difference was discernible in the nuclear soluble fraction between SIRT1-FL and SIRT1-ΔExon8. (0.96 MB TIF) [file pone.0013502.s009.tif]

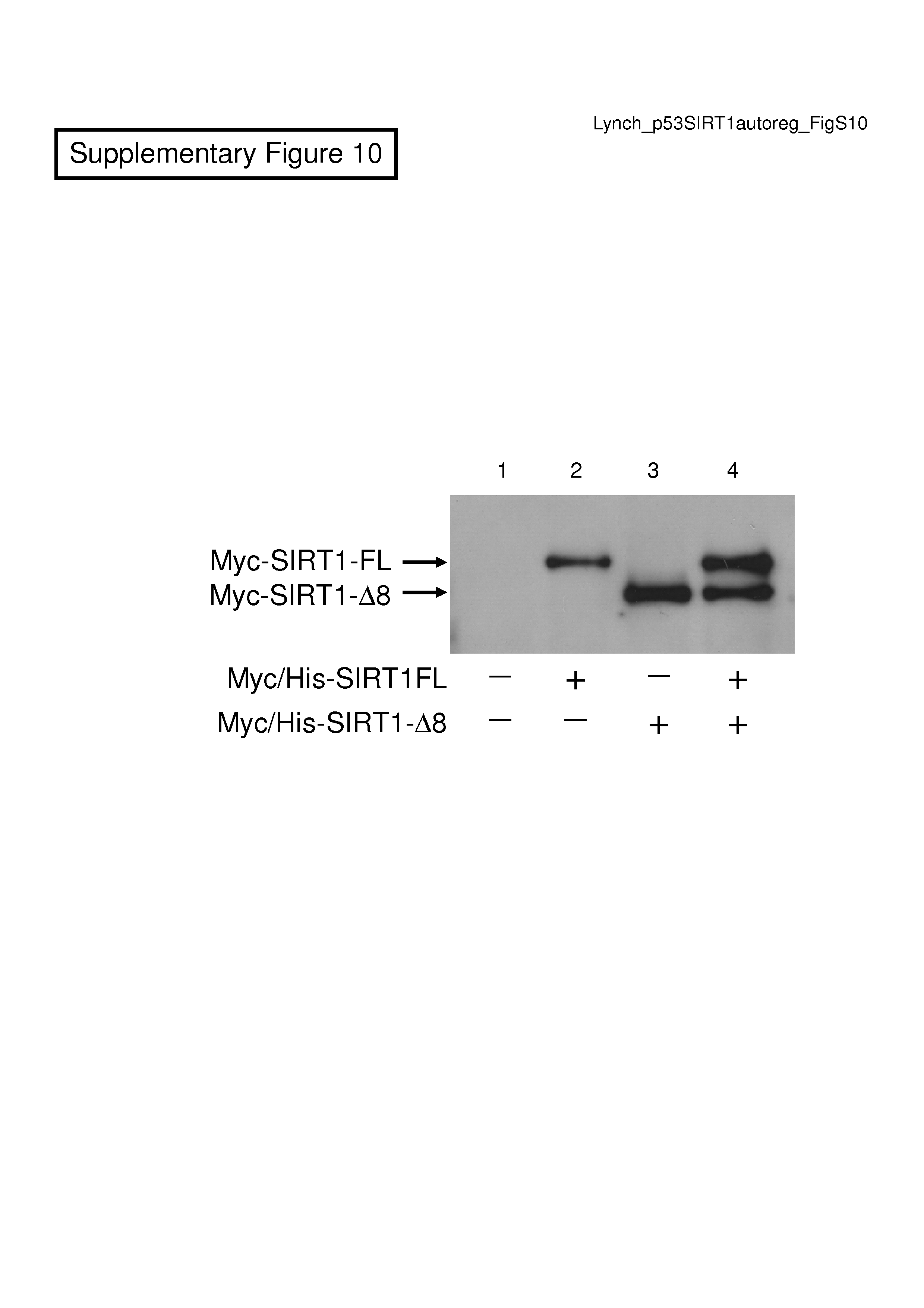

Supplement: Figure S10 — Analysis of the expression levels of purified His-SIRT1-FL and His-SIRT1-ΔExon8 for use in the deacetylase assay. HCT116 cells were transfected with the constructs indicated, followed by His-tag immunoprecipitation via Ni-Agarose columns as described in Methods. The levels of SIRT1-FL and SIRT1-ΔExon8 in the eluates were analysed by SDS-PAGE and blotting for the c-MYC tag. (0.91 MB TIF) [file pone.0013502.s010.tif]

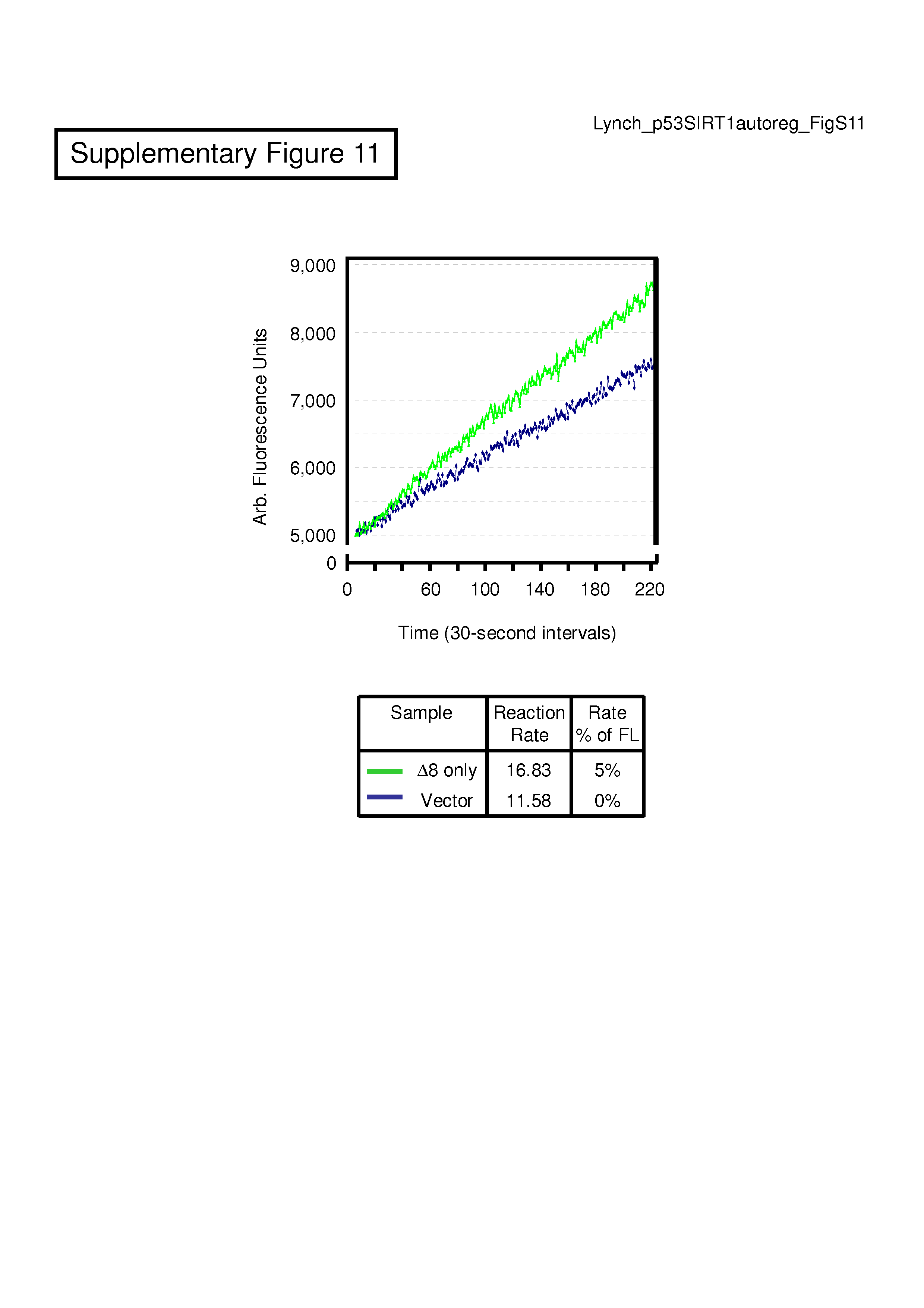

Supplement: Figure S11 — SIRT1-ΔExon8 has weak deacetylase activity in vitro. HCT116 cells were transfected with empty vector or SIRT1-ΔExon8, followed by His-tag immunoprecipitation via Ni-Agarose columns as described in Methods(see also: Supplementary Figure 10). This data is reproduced from Figure 6A, but here only SIRT1-ΔExon8 and the vector-only control are shown. Deacetylase activity of purified SIRT1-ΔExon8 protein was analysed by the fluorometric deacetylase assay and was greater than the background signal in the vector-only control. The table shows the reaction rate as a percentage of the deacetylase activity of SIRT1-FL (see: Figure 6A). The reaction rate was the slope of the linear regression best-fit line to each curve during the linear phase of the reaction. (0.68 MB TIF) [file pone.0013502.s011.tif]

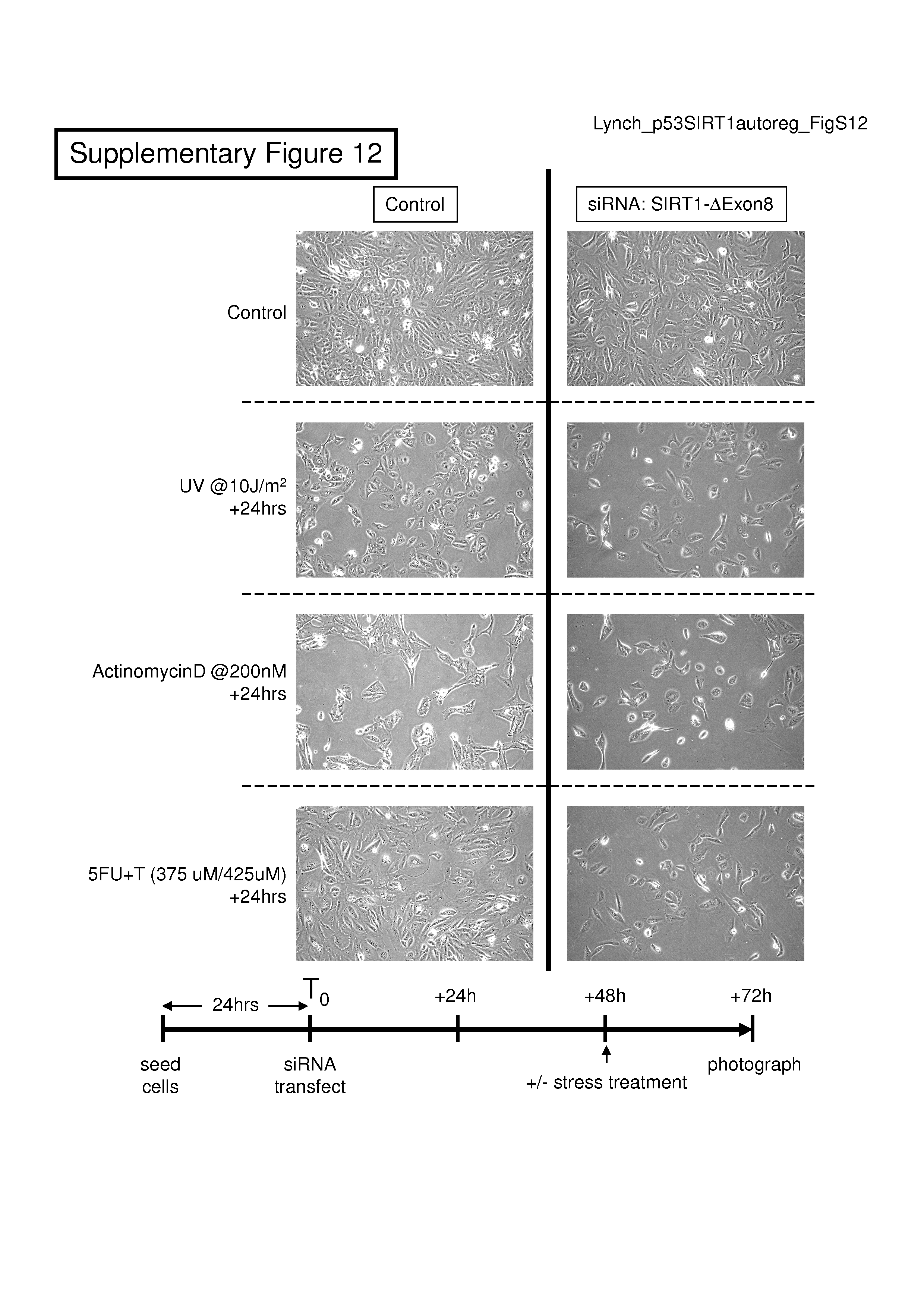

Supplement: Figure S12 — SIRT1-ΔExon8 displays an anti-apoptotic role after stress. ARPE19 cells were treated with siRNA for 48 hrs, then mock-treated or exposed to stress as indicated. Photographs taken +24 hrs after stress exposure. (3.82 MB TIF) [file pone.0013502.s012.tif]

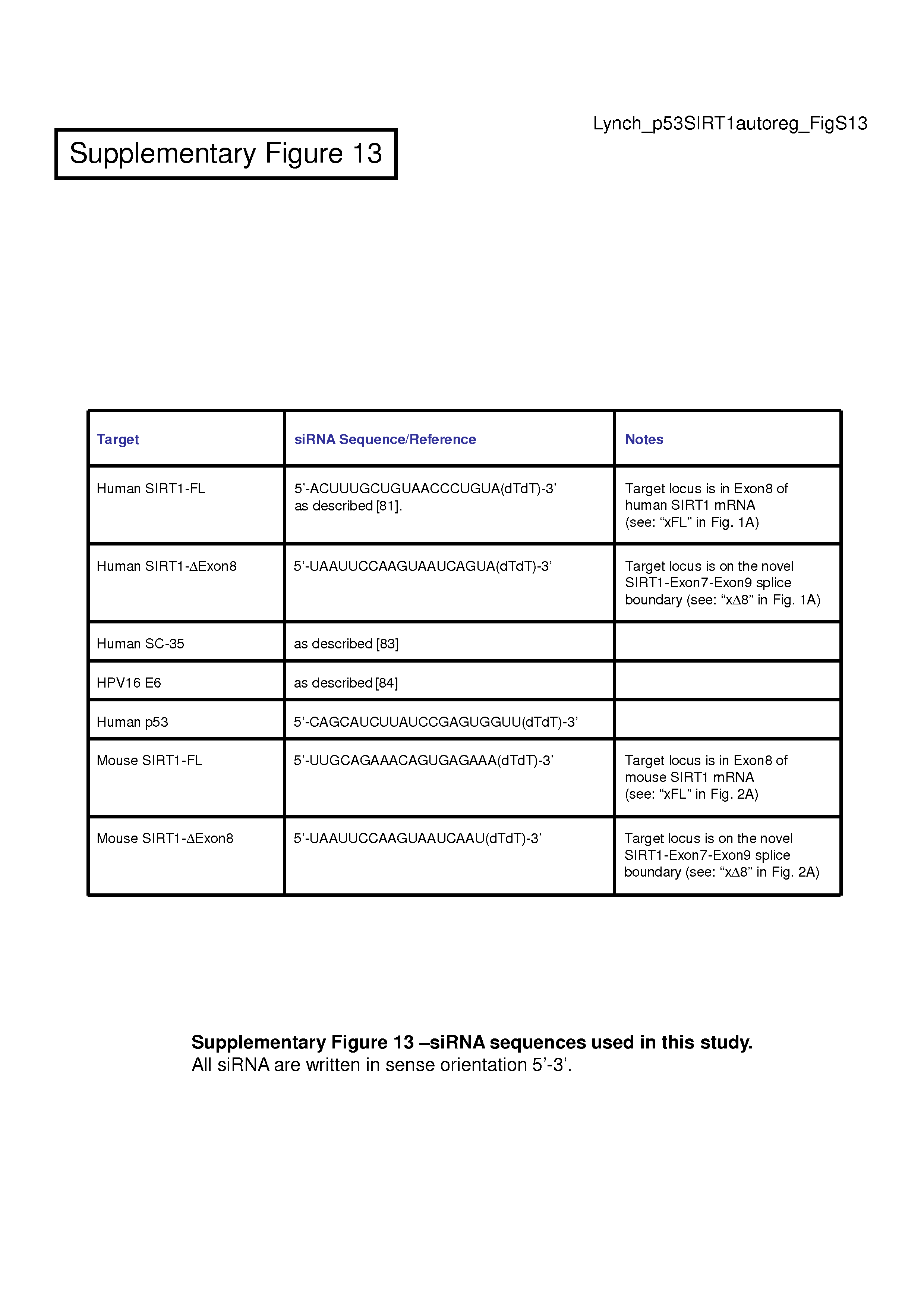

Supplement: Figure S13 — siRNA sequences used in this study. All siRNA are written in sense orientation 5′-3′. (0.72 MB TIF) [file pone.0013502.s013.tif]

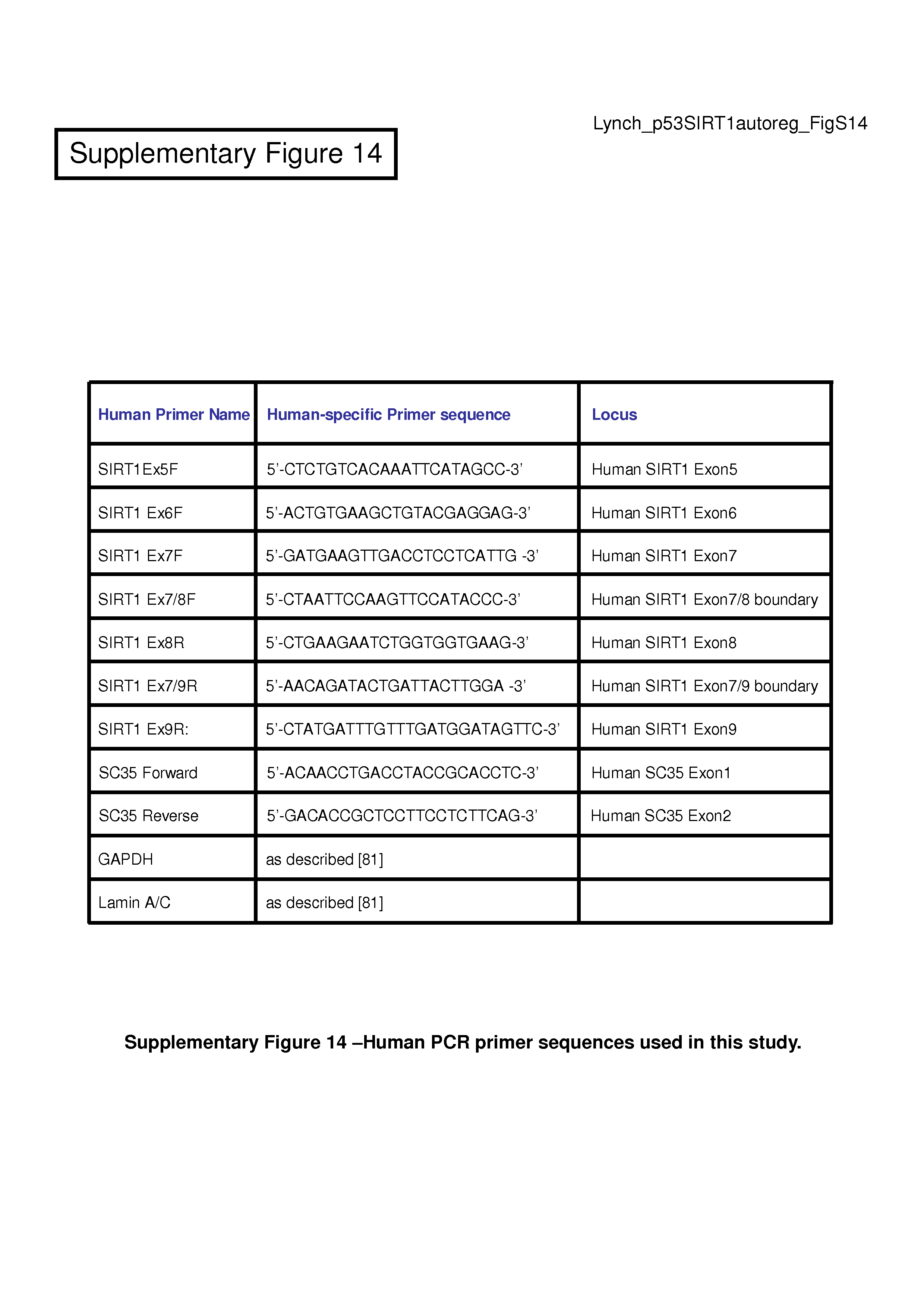

Supplement: Figure S14 — Human PCR primer sequences used in this study. (0.73 MB TIF) [file pone.0013502.s014.tif]

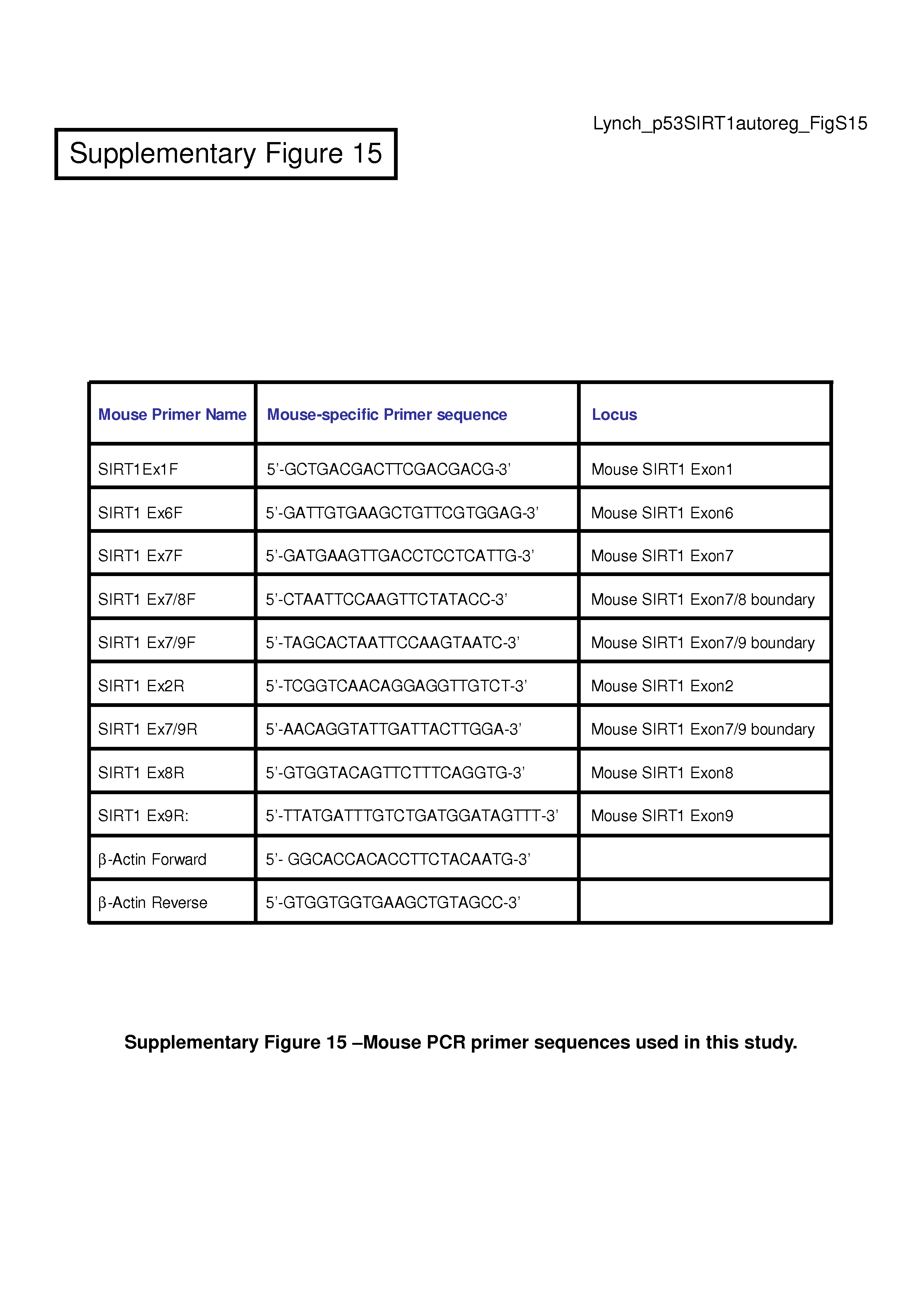

Supplement: Figure S15 — Mouse PCR primer sequences used in this study. (0.73 MB TIF) [file pone.0013502.s015.tif]

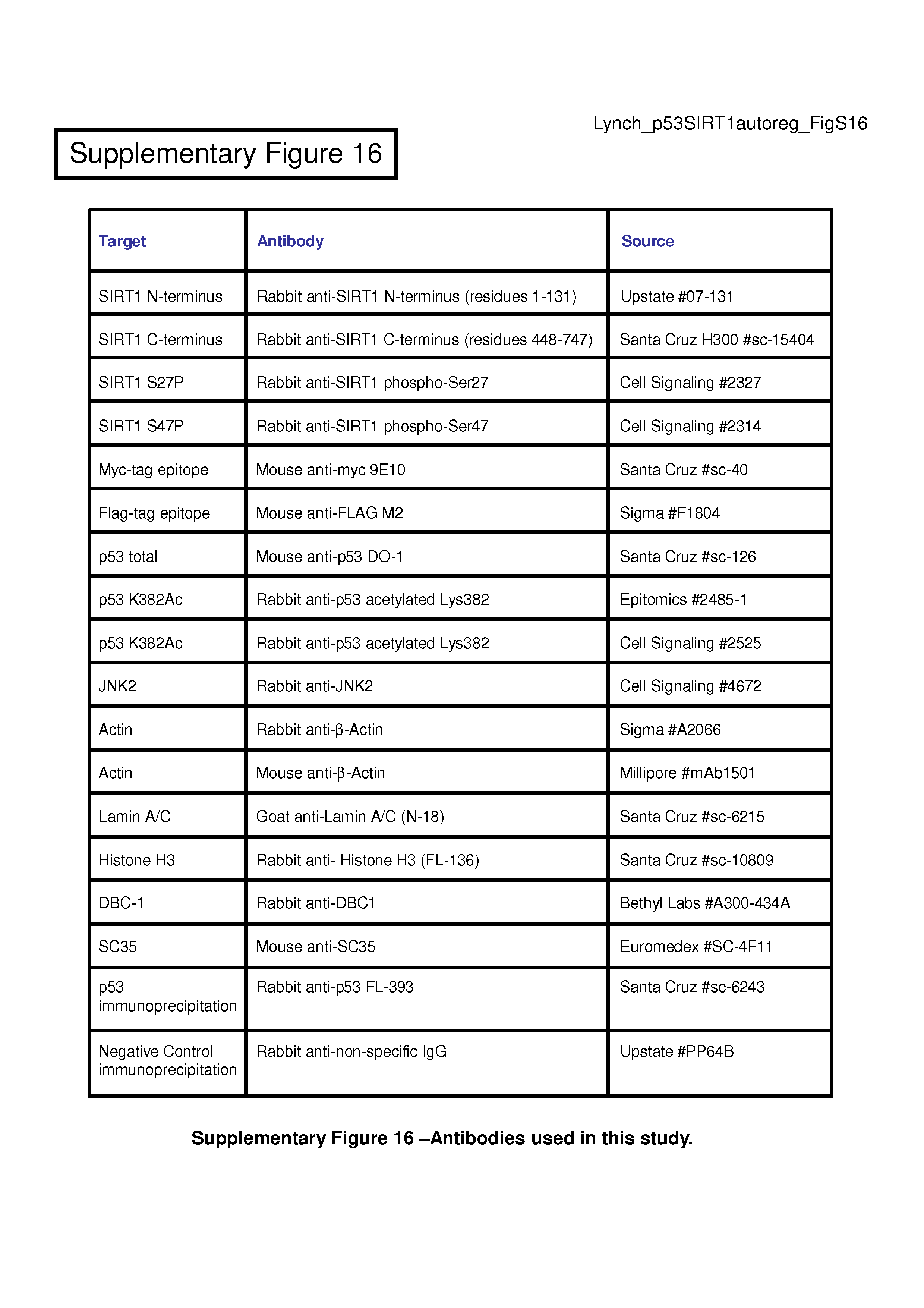

Supplement: Figure S16 — Antibodies used in this study. (0.79 MB TIF) [file pone.0013502.s016.tif]
